# Comparative efficacy and tolerability of front-line treatments for newly diagnosed chronic-phase chronic myeloid leukemia: an update network meta-analysis

**DOI:** 10.1186/s12885-019-6039-9

**Published:** 2019-08-28

**Authors:** Lu Tang, Huan Zhang, Yi-zhong Peng, Cheng-gong Li, Hui-wen Jiang, Min Xu, Heng Mei, Yu Hu

**Affiliations:** 1 Institute of Hematology, Union Hospital, Tongji Medical College, Huazhong University of Science and Technology, 1277 Jiefang Road, Wuhan, 430022, Hubei China; 2 Hubei clinical medical center of cell therapy for neoplastic disease, Wuhan, Hubei China; 3 Collaborative Innovation Center of Hematology, Huazhong University of Science and Technology, Wuhan, Hubei China; 4 Instisute of Pancreatic Surgery, Union Hospital, Tongji Medical College, Huazhong University of Science and Technology, 1227 Jiefang road, Wuhan, 430022, Hubei China; 5 Instisute of Orthopedics, Union Hospital, Tongji Medical College, Huazhong University of Science and Technology, 1227 Jiefang road, Wuhan, 430022, Hubei China

**Keywords:** Chronic myeloid leukemia, Network meta-analysis, Efficacy, Tolerability, Tyrosine kinase inhibitors

## Abstract

**Background:**

Recent years have witnessed the rapid evolution of therapies in chronic-phase chronic myeloid leukemia (CP-CML). To assess the efficacy and tolerability of all reported front-line treatments for patients with newly diagnosed CML, a multiple-treatments meta-analysis was performed, which accounted for both direct and indirect comparisons among those treatments.

**Methods:**

Primary outcomes were the percentage of patients achieving major molecular response (MMR) and complete cytogenetic response (CCyR) within 12 months. Secondary outcomes included the percentage of progression to accelerated phase (AP), serious adverse effects (AEs), overall discontinuation and discontinuation for drug-related AEs. Direct pairwise meta-analysis and indirect multi-comparison meta-analysis among those treatments in each outcome were both conducted. The surface under the cumulative ranking curve (SUCRA) was calculated for all treatments in each outcome. Cluster analysis demonstrated the division of treatments into distinct groupings according to efficacy and tolerability profiles.

**Results:**

A total of 21 randomized controlled trials (RCTs, including 10,187 patients) comparing 15 different interventions for CP-CML patients were included in this study. SUCRA analysis suggested that all tyrosine kinase inhibitors (TKIs) are highly effective in newly diagnosed CP-CML when compared to traditional drugs. Newer TKIs and higher-dose imatinib generally resulted in faster cytogenetic and molecular responses when compared with standard-dose imatinib and traditional drugs. Furthermore, traditional drugs, higher-dose imatinib and newer TKIs demonstrated lower acceptability than standard-dose imatinib. One cluster of interventions, which included nilotinib (300/400 mg BID), dasatinib (100 mg QD) and radotinib (300 mg BID), demonstrated higher efficacy and tolerability than other treatments.

**Conclusions:**

Nilotinib (300/400 mg BID), dasatinib (100 mg QD) and radotinib (300 mg BID) prove to be the most recommended front-line treatments of the greatest efficacy and tolerability for CP-CML patients. High-dose therapies are recommended only for patients in accelerated phase/blast phase or with suboptimal CML-CP response, and management of adverse events should be carried out to avoid compromising the clinical efficacy.

**Electronic supplementary material:**

The online version of this article (10.1186/s12885-019-6039-9) contains supplementary material, which is available to authorized users.

## Background

Chronic myeloid leukemia (CML) is one specific category of myeloproliferative neoplasm (MPN), characterized by an excessive proliferation of moderately and well differentiated cells of the granulocytic lineage [1]. The molecular abnormity of CML is the presence of an abnormal Philadelphia (Ph) chromosome, formed by a reciprocal translocation between the long arms of chromosomes 9 (ch9) and 22 (ch22). Central pathogenesis of CML is the fusion of the Abelson murine leukemia (ABL1) gene on ch9 with the breakpoint cluster region (BCR) gene on ch22, which results in expression of an oncoprotein termed BCR/ABL1 [2]. Compared to wild-type C-ABL1, BCR/ABL1 fusion protein displays increased kinase activity, which makes it a necessary and sufficient initiating trigger in CML [3]. According to conservative statistics, CML accounts for approximately 15% of adult leukemia, with an annual incidence of 1–2 cases per 100,000 persons. The diagnostic criteria, clinical characteristics, and natural course of the disease have been well defined in recent evidence-based guidelines for the diagnosis and management of CML [4]. The majority of diagnoses are made in the chronic phase (CP-CML) as opposed to the accelerated phase (AP-CML), therefore it is of great importance to confirm best front-line treatments in newly diagnosed CP-CML.

Before 2000, while the allogeneic stem cell transplant (Allo-SCT) offered greater chance of long-term survival, the mainstay of treatment for individuals ineligible for transplant was limited to interferon-alfa (IFN-α), busulfan, hydroxyurea (Hu) or chemotherapy [3, 5]. IFN-α led to disease regression and improved survival but was hindered by its limited efficacy and associated significant toxicities. Allo-SCT is curative, but carries great risks of mortality. In recent years, the CML therapeutic landscape has changed dramatically with the development of the small molecule tyrosine kinase inhibitors (TKIs) that potently interfered with the interaction between the BCR/ABL1 oncoprotein and adenosine triphosphate (ATP), blocking cellular proliferation of the malignant clone. This “targeted” approach altered the natural history of CML, improving the 10-year survival rate from approximately 20 to 80%–90% [6].

The previous systematic reviews and meta-analyses performed a direct comparison of the relative efficacy of two or more kinds of tyrosine kinase inhibitors for newly diagnosed CP-CML [7, 8]. Hofmann’s meta-analysis compared the major molecular response during the first year of standard-dose imatinib and high-dose imatinib or second-generation TKIs for chronic myeloid leukemia [9]. Yun’ study compared the outcomes of new generation TKIs versus imatinib in patients with newly diagnosed CP-CML, and concluded that new generation TKIs resulted in a greater major molecular response [10]. Chen’ group [11] conducted a network meta-analysis (NMA) of first-line treatments for CP-CML, and Fachi’ study [12] performed a NMA to compare the efficacy and safety of several TKIs. Although the previous studies conducted direct or indirect comparison among different therapies in CP-CML, none of them made a comprehensive comparison of all reported treatments, including conventional drugs, imatinib and new TKIs. Additionally, the dose difference of each drug may result in variation in efficacy. More importantly, the relative risks of serious adverse effect and treatment discontinuation should also be taken into consideration when we evaluate each kind of therapy. Herein, our study was the first meta-analysis that was based on multiple treatments to simultaneously assess the comparative efficacy and tolerability of almost all front-line treatments for newly diagnosed CML patients.

## Methods

This multiple comparison NMA was conducted in accordance with the recommendations of the Cochrane Comparing Multiple Interventions Methods Group [13] and the Preferred Reporting Items for Systematic Reviews and Meta-Analyses (PRISMA) extension statement for systematic reviews incorporating NMAs [14].

### Literature search

Two authors (Tang and Mei) independently used the following tools: MEDLINE, EMBASE, Cochrane library databases and ClinicalTrials.gov website to obtain relevant articles published until now. Following the PICOS principle (Participants, Interventions, Comparisons, Outcomes and Study design), the key search terms included “chronic myeloid leukemia, treatment, efficacy, safety, imatinib, nilotinib, bosutinib, dasatinib, radotinib, ponatinib, interferon, cytarabine, chemotherapy”. The complete search used for PubMed was: (((((((((((( chemotherapy [Title/Abstract]) OR cytarabine [Title/Abstract]) OR interferon [Title/Abstract]) OR ponatinib [Title/Abstract]) OR radotinib [Title/Abstract]) OR dasatinib [Title/Abstract]) OR bosutinib [Title/Abstract]) OR nilotinib [Title/Abstract]) OR imatinib [Title/Abstract]) OR treatment [Title/Abstract])) AND ((tolerability [Title/Abstract]) OR (efficacy [Title/Abstract])) AND chronic myeloid leukemia [Title/Abstract] Sort by: Best Match Filters: Clinical Trial; Humans. All eligible studies were considered for this review, and we also did a manual search, using the reference lists of key articles published.

### Outcome measures and eligibility criteria

Primary outcomes were the percentage of patients achieving major molecular response (MMR) and complete cytogenetic response (CCyR) within 12 months. Secondary outcomes included the percentage of progression to accelerated phase (AP), serious adverse effects (AEs in 3 or 4 grade), overall discontinuation and discontinuation for drug-related AEs. MMR is defined as achieving a ratio of BCR-ABL1 ≤ 0.1% on the international scale (≤ 0.1% BCR-ABL1[IS]) measured by reverse transcription-quantitative polymerase chain reaction (RT-qPCR) or ≥ 3-log reduction in BCR-ABL1 mRNA from the standard baseline if RT-qPCR is not available [15]. CCyR is defined as achieving 0% Philadelphia chromosome-positive (Ph+) metaphases by cytogenetic analysis of bone marrow [16]. Two researchers (Tang and Mei) independently assessed all the included studies and extracted the data. Studies were considered eligible if they met all the following inclusion criteria: (1) randomized controlled trials (RCTs) comparing at least two treatments as first line treatment for newly diagnosed, previously untreated (except for treatment with hydroxyurea or anagrelide) CP-CML patients; (2) the diagnosis of CML according to the trials was based on cytogenetic, fluorescence in situ hybridization (FISH) and/or RT-qPCR results; (3) sample size ≥40; (4) sufficient follow-up data about the above outcomes. When there were several reports concerning the same study, we included the high quality and most recent publication in our meta-analysis. Disagreements between the two reviewers were resolved by discussion with another reviewer (Hu).

### Assessment of risk of bias

As for quality assessment, the following domains were taken into consideration: random sequence generation, allocation concealment, blinding (self-reported), blinding (objective outcomes), incomplete and selective outcome reporting, and other bias presence. We made critical assessment separately for each domain and graded it as low risk for bias, unclear risk, or high risk for bias according to the criteria specified in the Cochrane Handbook [17].

### Data extraction

Data extraction was independently performed by two researchers (Tang and Mei), and any disagreement was resolved by a third researcher (Hu). For each RCT, the following characteristics were collected: the first author; publication year; trial number; study design, number of patients in each arm; interventions, gender and age distribution in participants, CML scoring systems (including Sokal risk and Hasford risk), ECOG (Eastern Cooperative Oncology Group) performances status and any relevant outcomes in this meta-analysis.

### Data synthesis and analysis

We produced visual inspection of separate network diagrams to show the amount of evidence available for each outcome in STATA v15.0. In each network plot, the size of each node is proportional to the total number of randomized participants (sample size) allocated to the corresponding treatment across all trials, and the width of each line is proportional to the total number of RCTs evaluating the corresponding treatment comparison. Odds ratios (ORs) with corresponding 95% confidence intervals (95% CIs) were calculated for dichotomous outcomes.

First, the pairwise meta-analysis was conducted to compare the same interventions to incorporate the assumption that the different studies were estimating different, yet related, treatment effects. Statistical heterogeneity was examined using the Cochran’s Q-statistic and a P-value of less than 0.01 was considered significant. I^2^ test was also used to quantify heterogeneity (ranging from 0 to100%). *P* < 0.01 for Q-test or I^2^ > 50% indicated the existence of heterogeneity across the studies. To minimizes the effect of heterogeneity, random-effect model was used. All statistical analysis in traditional meta-analysis was conducted using STATA v15.0.

Additionally, we made inferences between two intervention arms, such as A versus B, from indirect evidence (from combining studies through another intermediate comparator C) [18]. Network Meta-Analysis (NMA) is a technique to meta-analyze more than two interventions at the same time. Using a full Bayesian evidence network, all indirect comparisons are conducted to arrive at a single, integrated, estimate of the effect of all included treatments based on all included studies. Thus, even if there are no known comparisons for the investigated intervention, a network meta-analysis still can estimate the potential effect of this intervention based on existing head-to-head trials. We performed this network meta-analysis with a random-effects model based on a Bayesian framework using Markov Chain Monte Carlo methods in WinBUGS and R v3.0.2. To rank the treatments based on efficacy and safety, a probabilistic analysis was performed to estimate rank probabilities based on NMA, and the rank probabilities were summarized for each intervention in order to obtain the surface under the cumulative ranking curve (SUCRA). SUCRA analysis could illustrate the outcome percentages of every treatment relative to an ideal treatment, which always ranks first without uncertainty. The inconsistency refers to disagreements between direct and indirect evidence [19], and was estimated by the node-splitting method which generates *P* values for the null hypothesis that there is no significant inconsistency between direct and indirect evidence [20]. In case of significant inconsistency, we investigated the distribution of clinical and methodological variables that we suspected might be potential sources of either heterogeneity or inconsistency in every comparison-specific group of trials.

Finally, we produced a clustered ranking plot including SUCRA value for efficacy on the x-axis and SUCRA value for tolerability on the y-axis. Cluster analysis demonstrated the division of treatments into distinct groupings according to efficacy and tolerability profiles.

## Result

### Study characteristics and risk of bias assessment

**Fig. 1:**
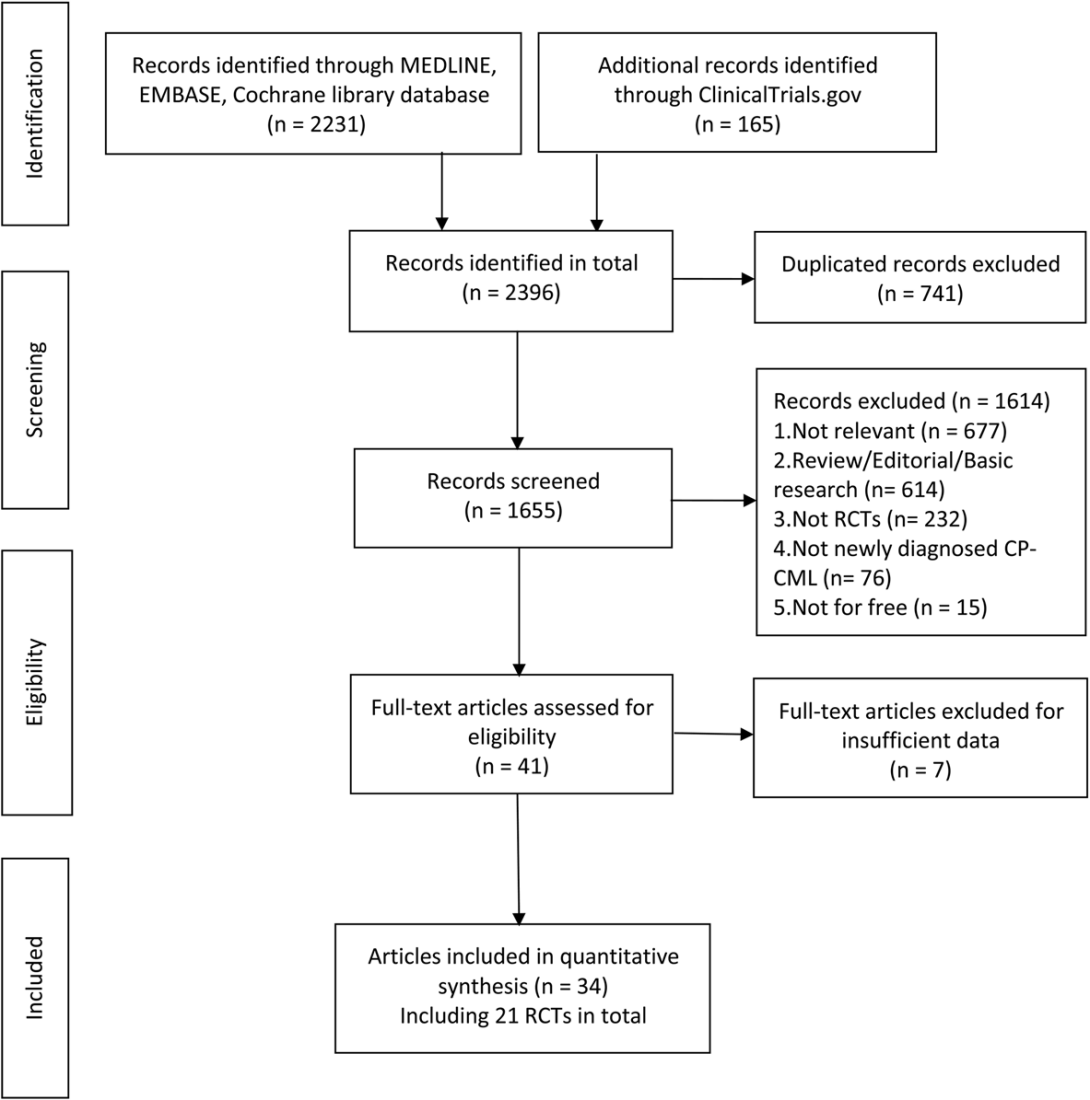
Flow diagram of selecting relevant published RCTs regarding front-line treatments in newly diagnosed CP-CML

A total of 2231 records were identified through the primary search, combined with additional 165 studies searched through ClinicalTrials.gov website (Fig. 1). Within these 2396 references, 741 were identified as ineligible due to duplication, leaving 1655 studies for selection, of which 1614 proved ineligible on the basis of titles, abstracts and full-text screening, leaving 34 eligible studies. 21 RCTs from 34 articles [21–54] involving 10,187 newly diagnosed CP-CML patients were included in this network meta-analysis. The characteristics of the included trials are summarized and presented in Table 1 and Additional file 1: Table S1. 20 trials (95.24%) described an adequate random sequence generation, and adequate treatment allocation concealment in 18 trials (85.71%). Double-blind (patients and treatment executors) strategies were carefully performed in 15 trials (71.42%), and blind strategies for objectively outcome assessors were involved in 20 trials (95.24%). The detailed assessment of the risk of bias is provided in Additional file 1: Table S2 and Additional file 2: Figure S1.

**Table 1:** Summary characteristics for the 21 eligible RCTs (10,187 patients)

Study Year	Journal Ref	Trial Number	Study Design	Treatment	Patients (N)	Male (%)	Age (yr)	Risk Group (%)					
								Sokal risk			Hasford risk		
								low	mid	high	low	mid	high
O’Brien (1) 2003	N Engl J Med [21–23]	NCT 00006343	Phase III, randomized, open-label, multicenter (IRIS)	Imatinib 400 mg qd	553	56.0	50(18–70)	53	29	18	46	44	10
				IFN-α + Ara-C	553	61.0	51(18–70)	47	30	22	45	45	10
Cortes (1) 2009	J Clin Oncol [24]	NCT 00124748	Phase III, randomized, multicenter	Imatinib 800 mg qd	319	57.4	48(18–75)	42.3	34.8	23.0	/	/	/
				Imatinib 400 mg qd	157	53.5	45(18–75)	39.5	33.8	27.0	/	/	/
Baccarani 2009	Blood [25]	NCT 00514488	Randomized, multicenter	Imatinib 800 mg qd	108	55.0	51(18–84)	/	/	/	/	/	/
				Imatinib 400 mg qd	108	57.0	51(18–81)	/	/	/	/	/	/
Preudhomme 2010	N Engl J Med [26]	NCT 00219739	Phase III, randomized (SPIRIT)	Imatinib 400 mg qd	159	69.0	50	38	38	24	/	/	/
				Imatinib 600 mg qd	160	56.0	51	37	38	28	/	/	/
				Imatinib 400 mg qd + Ara-C	158	58.0	55	37	41	23	/	/	/
				Imatinib 400 mg qd + IFN-α	160	65.0	51	36	40	24	/	/	/
Saglio 2010	N Engl J Med [27–30]	NCT 00471497	Phase III, randomized, open-label, multinational, multicenter (ENESTnd)	Nilotinib 300 mg bid	282	56.0	47(18–85)	37	36	28	/	/	/
				Nilotinib 400 mg bid	281	62.0	47(18–81)	37	36	28	/	/	/
				Imatinib 400 mg qd	283	56.0	46(18–80)	37	36	28	/	/	/
Kantarjian 2010	N Engl J Med [31–35]	NCT 00481247	Phase III, randomized, multicenter (DASISION)	Dasatinib 100 mg qd	259	56.0	46(18–84)	/	/	/	33	48	19
				Imatinib 400 mg qd	260	63.0	49(18–78)	/	/	/	33	47	19
Petzer 2010	Haematologica [36]	NCT 00327262	Randomized, multinational, multicenter (ISTAHIT)	Imatinib 800 mg qd	113	46.5	46(18–76)	/	/	/	/	/	/
				Imatinib 400 mg qd	113	42.5	46(20–68)	/	/	/	/	/	/
Cortes (2) 2011	Blood [37–39]	NCT 00574873	Randomized, multinational, multicenter (BELA)	Bosutinib 500 mg qd	250	60.0	48(19–91)	35	47	18	/	/	/
				Imatinib 400 mg qd	252	54.0	47(18–89)	35	47	18	/	/	/
Hehlmann (1) 2011	J Clin Oncol [40]	/	Randomized, multinational, multicenter	Imatinib 800 mg qd	338	59.0	52(18–86)	/	/	/	/	/	/
				Imatinib 400 mg qd	325	60.0	64(16–88)	/	/	/	/	/	/
				Imatinib 400 mg qd + IFN-α	351	61.0	54(16–83)	/	/	/	/	/	/
Radich 2012	Blood [41]	NCT 00070499	Phase III, randomized, multinational, multicenter	Dasatinib 100 mg qd	123	60.0	47(18–90)	/	/	/	36	33	32
				Imatinib 400 mg qd	123	59.0	50(19–89)	/	/	/	36	37	28
Thielen 2013	Ann Hematol [42]	NTR674	Phase III, randomized, multicenter	Imatinib 400 mg qd	55	NA	46(17–65)	29	44	22	/	/	/
				Imatinib 400 mg qd + Ara-C	54	NA	45(23–65)	37	39	20	/	/	/
Hughes 2014	Blood [43]	NCT 00760877	Phase III, randomized, open-label, multicenter (ENESTcmr)	Nilotinib 400 mg bid	104	68.3	46(23–82)	/	/	/	/	/	/
				Imatinib 400 mg qd	103	63.1	52(19–76)	/	/	/	/	/	/
O’Brien (2) 2014	Blood [44, 45]	/	Phase III, randomized, multinational, multicenter (SPIRIT2)	Dasatinib 100 mg qd	407	61.0	53(18–89)	/	/	/	/	/	/
				Imatinib 400 mg qd	407	60.0	53(18–87)	/	/	/	/	/	/
Deininger 2014	Br J Hematol [46]	NCT 00070499	Phase II, randomized	Imatinib 800 mg qd	73	64.0	52(19–82)	/	/	/	21	30	49
				Imatinib 400 mg qd	72	63.0	50(23–80)	/	/	/	21	30	49
Hjorth-Hansen 2015	Eu J Hematol [47]	NCT 00852566	Phase II, randomized multicenter (NordCML006)	Dasatinib 100 mg qd	22	32.0	53(29–71)	32	45	23	/	/	/
				Imatinib 400 mg qd	24	63.0	58(38–78)	49	34	17	/	/	/
Wang 2015	Blood [48]	NCT 01275196	Phase III, randomized, multicenter	Nilotinib 300 mg bid	134	68.0	41(18–76)	51	33	16	/	/	/
				Imatinib 400 mg qd	133	61.0	39(19–74)	52	32	16	/	/	/
Kwak 2015	Blood [49]	NCT 01511289	Phase III, randomized, open-label, multicenter (RERISE)	Radotinib 300 mg bid	79	66.0	45(20–75)	27	48	25	/	/	/
				Radotinib 400 mg bid	81	58.0	43(18–84)	27	48	25	/	/	/
				Imatinib 400 mg qd	81	64.0	45(18–83)	27	48	37	/	/	/
Lipton 2016	Lancet Oncol [50]	NCT 01650805	Phase III, randomized, open-label, multicenter	Ponatinib 45 mg qd	155	63.0	55(18–89)	41	41	17	/	/	/
				Imatinib 400 mg qd	152	61.0	52(18–86)	41	44	15	/	/	/
Cortes (3) 2016	Lancet Haematol [51]	NCT 00802841	Phase III, randomized, multicenter (LASOR)	Nilotinib 400 mg bid	96	56.0	46(32–46)	/	/	/	/	/	/
				Imatinib 600 mg qd	95	61.0	44(33–56)	/	/	/	/	/	/
Hehlmann (2) 2017	Leukemia [52, 53]	NCT 00055874	Randomized, open-label, multinational, multicenter	Imatinib 400 mg qd	400	61.0	53(16–88)	36	40	25	/	/	/
				Imatinib 400 mg qd + IFN-α	430	59.0	53(16–83)	39	39	22	/	/	/
				Imatinib 400 mg qd + Ara-C	158	63.0	52(18–79)	39	34	27	/	/	/
				Imatinib 400 mg qd after IFN-α	128	63.0	53(18–87)	40	45	15	/	/	/
				Imatinib 800 mg qd	420	59.0	51(18–85)	37	37	27	/	/	/
Cortes (4) 2018	J Clin Oncol [54]	NCT 02130557	Phase III, randomized, open-label, multicenter	Bosutinib 400 mg qd	268	57.7	52(18–84)	38	41	201	/	/	/
				Imatinib 400 mg qd	268	56.0	53(19–84)	40	39	21	/	/	/

(“/” means “not available”)

### Network geometry

**Fig. 2:**
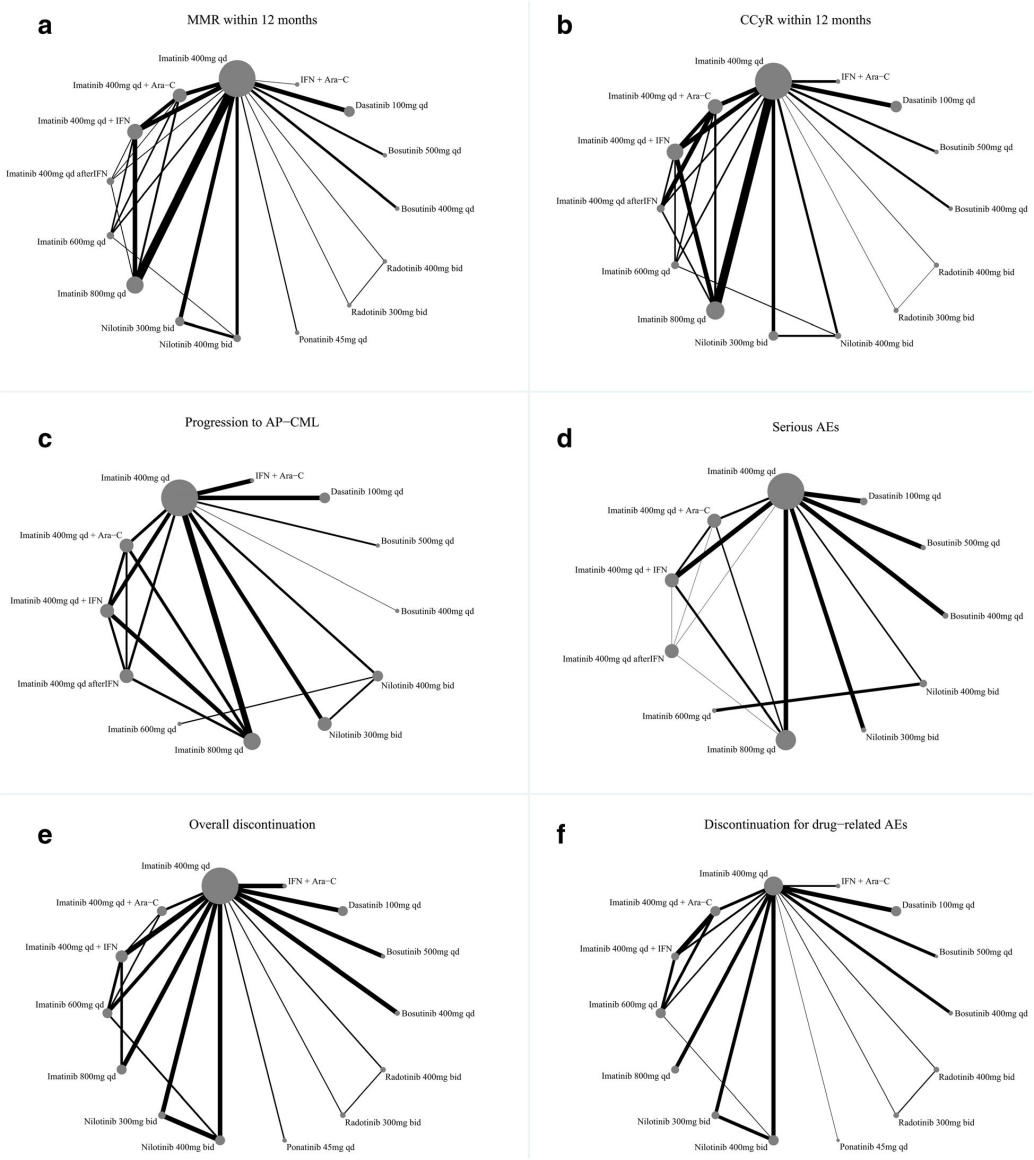
Network graphs of eligible trials assessing front-line treatments in newly diagnosed CP-CML for six outcomes. (**a**) MMR within 12 months; (**b**) CCyR within 12 months; (**c**) Progression to AP-CML; (**d**) Serious AEs; (**e**) Overall discontinuation; (**f**) Discontinuation for drug-related AEs

The six network graphical structures for each outcome display the available direct comparisons of the network of trials organized from the included RCTs (Fig. 2). The recommended standard treatment “imatinb 400 mg QD” were thoroughly compared against every other treatment. Novel drug, such as ponatinib, radotinib and bosutinib were only compared against standard treatment “imatinib 400mg QD”. MMR and CCyR within 12 months (Fig. 2a–b) were reported in almost all trials (21 RCTs including 10,187 patients and 19 RCTs including 9673 patients, respectively), progression to AP-CML and serous AEsc (Fig. 2c–d) were reported in quite limited trials (8 RCTs including 5712 patients and 10 RCTs including 4152 patients, respectively), whereas overall discontinuation and discontinuation for drug-related AEs (Fig. 2e–f) were both reported in 18 trials (8209 and 7411 patients, respectively).

### Direct pairwise meta-analysis

All treatments had at least one comparison with the standard treatment “imatinib 400 mg QD”, and several of them were directly compared with two or more other treatments (Additional file 1: Table S3). As for efficacy, newer TKIs, such as dasatinib, radotinib, bosutinib, nilotinib and ponatinib, showed higher efficacy than imatinib in the first-line treatment of CP-CML patients, but the traditional treatment, such as IFN-α and Ara-C, suggested significantly lower efficacy when compared to TKIs. Low-dose nilotinib (300 mg BID) and radotinib (300 mg BID) had higher efficacy than high-dose nilotinib (400 mg BID) and radotinib (400 mg BID), respectively. For overall discontinuation, traditional drugs showed higher dropout rate than TKIs. As for the discontinuation specially caused by drug-related adverse effects, most treatments showed lower acceptability when compared to standard-dose imatinib (400 mg QD), such as traditional drugs, newer TKIs and higher-dose imatinib (600 or 800 mg QD). However, low-dose nilotinib (300 mg QD) generated higher tolerability than standard-dose imatinib (400 mg QD). Moreover, standard-dose imatinib (400 mg QD) showed least probability of serious AEs when compared to other treatments. On the whole, statistical heterogeneity was moderate, although 95% CIs were wide for several comparisons, which portrayed the small number of studies available for the pair-wise comparison. Substantial heterogeneity was observed when comparing imatinib 400 mg QD with nilotinib 400 mg BID (I^2^ = 75.7%) for MMR or imatinib 400 mg QD + Ara-C (I^2^ = 87.3%) for CCyR. Nevertheless, there was no evidence showing heterogeneity in other pooled results of the direct comparisons for the six outcomes.

### Transitivity and consistency assessment

As there were no observed significant clinical differences in distribution of effect modifiers between trials comparing different sets of interventions, we considered that the transitivity assumption was almost met (see Table 1 and Additional file 1: Table S1). All closed loops (networks of three comparisons that arise when collating studies involving different selections of competing treatments) were consistent, since the 95% CIs of inconsistency factors (IF, the difference between the direct and indirect estimate for one of the comparisons in a particular loop) included zero. Furthermore, inconsistency test by the node-splitting method indicated that there was no significant inconsistency between direct and indirect evidence for nearly all *P* values were higher than 0.05 (Additional file 1: Table S4). Analysis of inconsistency indicated that there was inconsistency in the loop for “CCyR” (“imatinib 400 mg QD + Ara-C” - “imatinib 800 mg QD”), another loop for “discontinuation for drug-related AEs” (“imatinib 400 mg QD + Ara-C” - “imatinib 600 mg QD”) and none for other four outcomes. Furtherly, we identified slight gender and sex difference across comparisons in these two loops, which may account for the inconsistency.

### Network estimation and cumulative ranking

Pooled ORs with corresponding 95% CIs for the efficacy and tolerability of different treatments from the network meta-analysis are shown in Table 2 and Additional file 1: Table S5. Rankograms that show the distribution of the probabilities of every treatment being ranked at each of the possible are presented in Additional file 2: Figure S2, and Table 3 presents all SUCRA values in terms of both efficacy and acceptability of each intervention. As for primary outcomes in MMR and CCyR, higher-dose imatinib (600 or 800 mg QD) and newer TKIs, such as ponatinib, radotinib, bosutinib, nilotinib and dasatinib, were all highly effective in comparison to standard-dose imatinib, except that imatinib (600 mg QD) showed lower effective in CCyR. Obviously, the traditional treatment, such as IFN-α and Ara-C, generated significantly lower efficacy when compared to TKIs. Among newer TKIs, ponatinib was identified to be the most effective, and nilotinib, radotinib, dasatinib as well as bosutinib showed relatively higher efficacy. Nilotinib (300 or 400 mg BID), dasatinib (100 mg QD), low-dose bosutinib (400 mg QD) and higher-dose imatinib (600 or 800 mg QD) showed lower probability of disease progression to AP-CML. As for serious AEs, there were no significant difference among studied treatments, SUCRAs of which ranged from 0.477 to 0.632, except that SUCRA for imatinib 400 mg QD after IFN was 0.159. In terms of discontinuation for drug-related AEs, standard-dose imatinib (400 mg QD) was the most tolerable treatment, and nilotinib (300 or 400 mg BID), dasatinib (100 mg QD), higher-dose imatinib (600 or 800 mg QD), and low-dose radotinib (300 mg BID) were better than other treatments. Traditional drugs and newer TKIs showed lower acceptability than imatinib, and the drug toxicity were positively associated with drug dose. But as for overall discontinuation, low-dose radotinib (300 mg BID) suggested lowest treatment discontinuation, and imatinib (400 or 600 mg QD), nilotinib (300 or 400 mg BID), and low-dose bosutinib (400 mg QD) showed relatively lower dropout rate than other treatments.

**Table 2:**
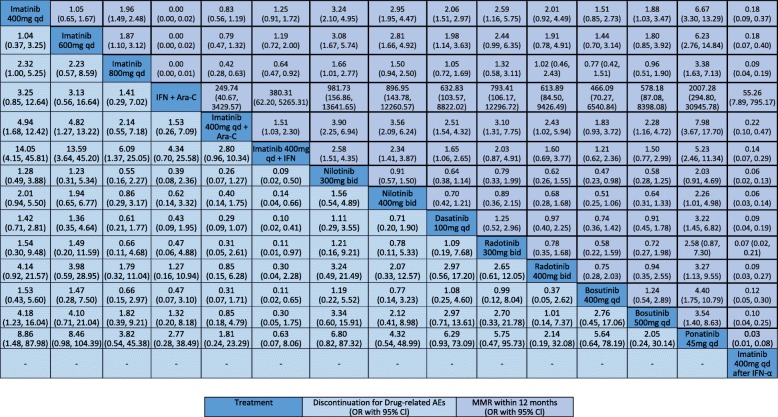
Efficacy and tolerability of all treatments for CP-CML according to Bayesian network meta-analysis

**Table 3:** Surface under the cumulative ranking curve (SUCRA) data for six outcomes

Treatment	Surface Under the Cumulative Ranking Curve (SUCRA)					
	MMR within 12 months	CCyR within 12 months	Progression to AP-CML	Overall Discontinuation	Discontinuation for Drug-related AEs	Serious AEs
Bosutinib 400 mg qd	0.428	0.684	0.259	0.318	0.346	0.572
Bosutinib 500 mg qd	0.570	0.416	0.872	0.520	0.719	0.581
Dasatinib 100 mg qd	0.624	0.736	0.302	0.337	0.312	0.632
IFN + Ara-C	0.000	0.000	0.719	0.726	0.476	–
Imatinib 400 mg qd	0.209	0.335	0.406	0.383	0.158	0.481
Imatinib 400 mg qd + Ara-C	0.172	0.340	0.482	0.903	0.769	0.483
Imatinib 400 mg qd + IFN	0.321	0.411	0.350	0.653	0.958	0.488
Imatinib 400 mg qd after IFN	0.071	0.076	–	–	–	0.159
Imatinib 600 mg qd	0.449	0.311	0.254	0.352	0.187	0.477
Imatinib 800 mg qd	0.682	0.730	0.388	0.525	0.576	0.528
Nilotinib 300 mg bid	0.791	0.759	0.301	0.372	0.234	0.543
Nilotinib 400 mg bid	0.839	0.781	0.293	0.416	0.440	0.556
Ponatinib 45 mg qd	0.997	–	0.419	0.649	0.837	–
Radotinib 300 mg bid	0.750	0.895	–	0.283	0.343	–
Radotinib 400 mg bid	0.596	0.538	–	0.561	0.688	–

(“-” means “can’t be evaluated”)

### Cluster analysis

**Fig. 3:**
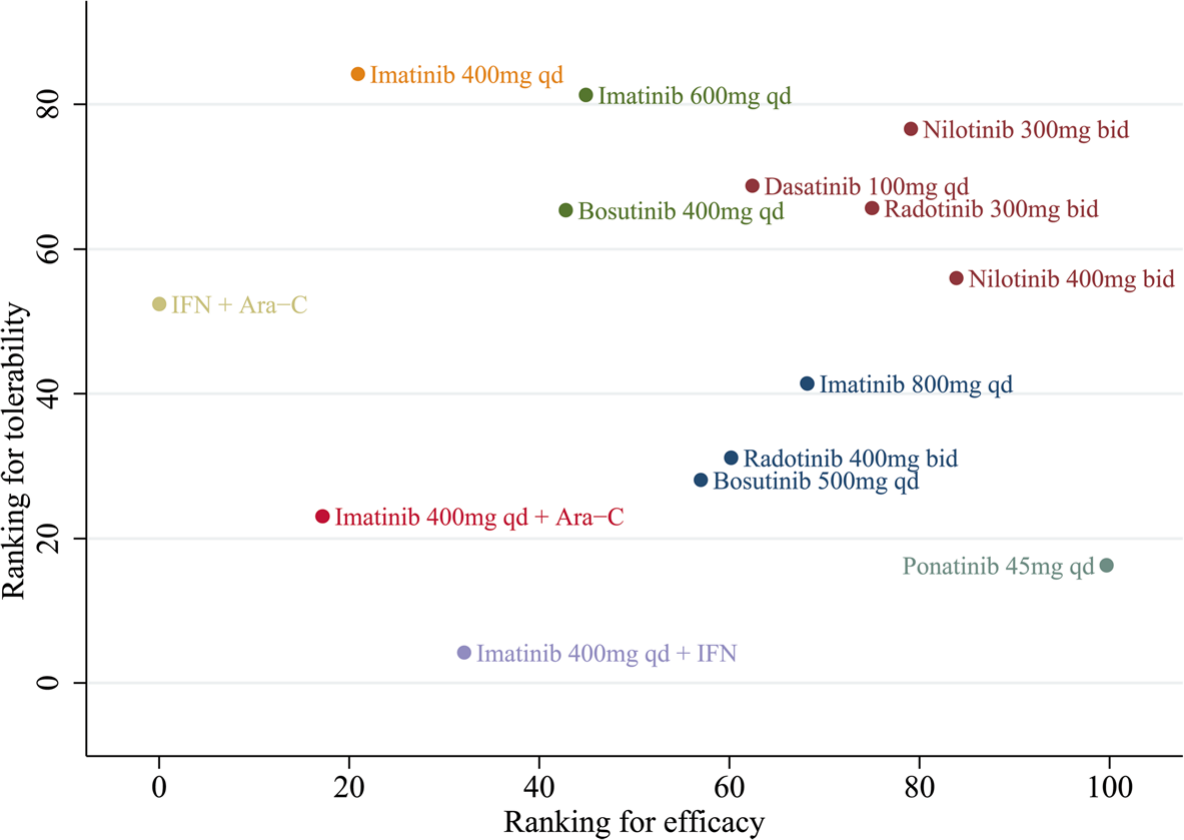
Comprehensive ranking (efficacy and tolerability) of front-line treatments in newly diagnosed CP-CML. (Efficacy is evaluated as MMR within 12 months and tolerability is evaluated as less discontinuation for drug-related AEs)

Utilizing the SUCRA values, we displayed a clustered ranking plot of these treatments in the two dimensions of the x-axis (efficacy as higher MMR within 12 months) and the y-axis (tolerability as less discontinuation for drug-related AEs) in Fig. 3. Cluster analysis demonstrated the division of treatments into eight distinct groups. One cluster of interventions, which includes nilotinib (300 or 400 mg BID), radotinib (300 mg BID) and dasatinib (100 mg QD), has relatively higher efficacy and tolerability compared with other treatments. Ponatinib (45 mg QD) and imatinib (400 mg QD) suggested highest efficacy and tolerability, respectively.

### Reporting bias

The funnel plots seemed to be approximately symmetrical for four outcomes (MMR, CCyR, progression to AP-CML and overall discontinuation), but rather asymmetrical for serious AEs and discontinuation for drug-related AEs, which suggests that several studied treatments were favored more in small trials (Additional file 2: Figure S3).

## Discussion

To our knowledge, this was the first to comprehensively assess the comparative efficacy and tolerability of almost all front-line treatments for newly diagnosed CP-CML patients, involving 21 RCTs (10,187 patients). Our study suggests both statistically and clinically significant differences among front-line treatments of newly diagnosed CP-CML patients.

Regarding the efficacy, we focused on three important indicators, early major molecular response (MMR, ≤0.1% BCR-ABL1[IS]), complete cytogenetic response (CCyR, ≤1% BCR-ABL1[IS]) and disease progression to AP-CML. The prognostic significance of early MMR and CCyR after first-line treatment has been evaluated in several studies [55–57]. Achievement of MMR and CCyR within 12 months is an established prognostic indicator of long-term survival. Furthermore, achievement of MMR within 12 months is associated with a very low probability of subsequent disease progression and a high likelihood of achieving a subsequent deep molecular response. In addition, disease progression to AP-CML while on drug therapy usually has worse prognosis than de novo AP-CML. In terms of tolerability, we focused on serious AEs, overall discontinuation and discontinuation due to drug-related AEs during therapy at early stage. Serious AEs refer to adverse effects in higher (3 or 4) grade, including non-hematological and hematological adverse effects. Overall treatment discontinuation is influenced by many factors, including drug-related AEs, refusal, failure to achieve complete hematologic response, relapse and disease progression. However, as for discontinuation for drug-related AEs, it specifically refers to the safety of the therapeutic drug, which is more likely to reveal actual drug tolerability.

The treatment of CML has undergone an evolution with the advent of imatinib, which has significantly changed the natural history of the disease with an increase of 10-year OS from 10 to 20% to 80–90% [6]. According to our study, standard-dose imatinib (400 mg QD) proves to be of greater efficacy than traditional drugs or the combination therapy of imatinib with traditional drugs. However, several patients may have resistance and/or intolerance to imatinib and these patients require further treatment options, such as second-generation TKIs and ponatinib.

Our analysis suggests that patients receiving nilotinib as initial treatment achieve faster cytogenetic and molecular responses with a lower rate of transformation to more advanced phases of CML and relatively higher drug tolerability. Therefore, nilotinib might be an excellent choice as front-line therapy in CP-CML due to greater potency and selectivity for BCR-ABL1 kinase inhibition and higher tolerability. However, some observed long-term toxicity effects (particularly cardiovascular events and diabetes mellitus) suggest that nilotinib should be used with caution in patients with cardiovascular risk factors and metabolic syndrome [58]. Additionally, dasatinib (100 mg QD) and radotinib (300 mg BID) demonstrates almost similar efficacy and acceptability as nilotinib. Nilotinib, dasatinib and bosutinib are second-generation TKIs approved in many countries for CML following many international multicenter trials, but radotinib is currently approved only in Korea for this indication. High-dose imatinib (800 mg QD), radotinib (400 mg BID) and bosutinib (500 mg QD) demonstrates very low tolerability, thus they are recommended only for patients in accelerated phase/blast phase or with suboptimal CML-CP response. Management of adverse events should be carried out to avoid compromising the clinical efficacy. Ponatinib, the most recently approved TKI, was found to be of greatest probability of MMR within 12 months, but relatively higher tendency of treatment dropout. Ponatinib has demonstrated efficacy in patients with refractory CML, but is associated with an increased risk of arterial hypertension, sometimes severe, and serious arterial occlusive and venous thromboembolic events [16]. CML patients, with presence of the T315I mutation, resistance or intolerance to other TKIs, may be an appropriate candidate for ponatinib therapy [16, 59]. As mentioned before, TKI drugs that can achieve faster MMR are usually associated with low disease progression and high likelihood of achieving a deep molecular response. Our study suggests that newer-generation TKIs generate faster molecular and cytogenic response. The primary goal of TKI therapy for CP-CML patients is prevention of disease progression, which is more common in patients with intermediate- or high-risk score. Therefore, newer-generation TKI drugs with a low probability of disease progression are preferred for patients with intermediate or high risk. Almost all TKIs are more tolerated than traditional drugs, and the difference in their potential toxicity profiles may influence the selection of initial therapy. In terms of starting does of TKI drugs, only patients who can actually tolerate the potential drug toxicity are recommend to receive high-dose therapy.

The NCCN guideline recommends imatinib (400 mg QD) and second-generation TKIs (dasatinib (100 mg QD), nilotinib (300 mg BID) and bosutinib (400 mg QD) as appropriate options for front-line TKI therapy for patients with CP-CML across all risk scores [15]. Additionally, the previous two meta-analysis concluded that nilotinib seems to be the best choice for first-line therapy in CML patients, compared with the currently available TKIs on the international market [11, 12]. Unlike the previous study, our study introduced the analysis of new first-line TKI therapy, such as radotinib and ponatinib. One cluster of treatments including nilotinib (300 or 400 mg BID), radotinib (300 mg BID) and dasatinib (100 mg QD), demonstrates relatively greater efficacy and tolerability than other treatments. Although ponatinb suggests highest efficacy in early cytogenetic and molecular responses, its great side-effects and weak tolerability can’t be ignored. High-dose imatinib (800 mg QD), radotinib (400 mg BID) and bosutinib (500 mg QD) are recommended only for patients in accelerated phase/blast phase or with suboptimal CML-CP response. These results may have potential clinical implications, which provide useful information for clinical decision-making and should be considered in the development of clinical practice guidelines. But during the clinical practice, the selection of front-line TKI therapy should be based on several factors, such as risk score, patient’s stage, ability to tolerate therapy, drug toxicity and the present comorbid conditions [15].

Strictly speaking, we designed this NMA as standardized by the PRISMA principle and conducted it carefully to minimize errors and ensure the validity of findings from all eligible trials. Nevertheless, there are also several limitations to our research due to its nature or design. Firstly, we just included limited number of trials, and several drugs (such as radotinib, ponatinib) were only used in limited countries and areas. Secondly, although MMR and CCyR are important prognostic indicator, which are able to represent the disease prognosis to some degree, we were unable to perform the NMA of some important prognostic outcomes directly for insufficient follow-up data, such as overall survival (OS) and progression-free survival (PFS). Thirdly, we didn’t take the cost-effective element into consideration, thus further cost-effectiveness analyses are necessary to evaluate the economic feasibility. Fourthly, some estimated results of this NMA relied on indirect comparisons despite of no evidence suggesting obvious inconsistency. Therefore, the application of our study should take into account any limitations of the analysis and the specific clinical situation.

## Conclusions

In conclusion, one cluster of treatments including nilotinib (300 or 400 mg BID), radotinib (300 mg BID) and dasatinib (100 mg QD), might be an excellent choice as front-line therapy in CP-CML due to superior efficacy and tolerability than other treatments. High-dose TKI therapies are recommended only for patients in accelerated phase/blastic phase or with suboptimal CML-CP response, and management of adverse events should be carried out to avoid compromising the clinical efficacy. Results from our study have potential clinical implications, which provide useful information for clinical decision-making and should be considered in the development of clinical practice guidelines. In the future, more clinical trials will be necessary to investigate further role of TKI therapy in CP-CML patients. Furthermore, new TKI treatments with higher efficacy or acceptability than the existing treatments are urgently needed to be explored.

## Additional files

Additional file 1: **Table S1.** Supplementary characteristics of trials and patients in the 21 eligible RCTs (10,187 patients). **Table S2.** Risk of bias assessment. **Table S3.** Efficacy and tolerability of all treatments according to pairwise estimates. **Table S4.** Inconsistency evaluation. **Table S5.** Efficacy and tolerability of all treatments according to Bayesian network meta-analysis. Appendix 1 WinBUGS codes (PDF 263 kb)

Additional file 2: **Figure S1.** Risk of bias assessment (a) Risk of bias graph; (b) Risk of bias summary. **Figure S2.** Rankograms for six outcomes (a) MMR within 12 months; (b) CCyR within 12 months; (c) Progression to AP-CML; (d) Serious AEs; (e) Overall discontinuation; (f) Discontinuation for drug-related AEs. **Figure S3.** Funnel plots for six outcomes (a) MMR within 12 months; (b) CCyR within 12 months; (c) Progression to AP-CML; (d) Serious AEs; (e) Overall discontinuation; (f) Discontinuation for drug-related AEs (PDF 2981 kb)

## Data Availability

The authors declare that all data supporting the findings of this study are available within the article and the enrolled articles for meta-analysis.

## Abbreviations

ABL1: Abelson murine leukemia 1
AE: Adverse events
Allo-SCT: Allogeneic stem cell transplant
AP-CML: Accelerated-phase chronic myeloid leukemia
Ara-C: Cytosine arabinoside
ATP: Adenosine triphosphate
BCR: Breakpoint cluster region
CCyR: Complete cytogenetic response
CI: Confidence interval
CP-CML: Chronic-phase chronic myeloid leukemia
ECOG: Eastern Cooperative Oncology Group
FISH: Fluorescence in situ hybridization
Hu: Hydroxyurea
IF: Inconsistency factor
IFN-α: Interferon-alfa
MMR: Major molecular response
MPN: Myeloproliferative neoplasm
NCI: National Cancer Institute
NMA: Network meta-analysis
OR: Odds ratio
OS: Overall survival
PFS: Progression-free survival
Ph: Philadelphia chromosome
PRISMA: Preferred Reporting Items for Systematic Reviews and Meta-Analyses
RCT: Randomized controlled trial
RT-qPCR: Reverse transcription-quantitative polymerase chain reaction
SUCRA: Surface under the cumulative ranking
TKI: Tyrosine kinase inhibitors
