# Supplementary material for: Comparative efficacy and tolerability of front-line treatments for newly diagnosed chronic-phase chronic myeloid leukemia: an update network meta-analysis
Source: BMC Cancer. 2019 Aug 28;19:849. doi: 10.1186/s12885-019-6039-9 (PMC6714291; doi:10.1186/s12885-019-6039-9)
Supplement: Supplementary file 1 — Table S1. Supplementary characteristics of trials and patients in the 21 eligible RCTs (10,187 patients). Table S2. Risk of bias assessment. Table S3. Efficacy and tolerability of all treatments according to pairwise estimates. Table S4. Inconsistency evaluation. Table S5. Efficacy and tolerability of all treatments according to Bayesian network meta-analysis. Appendix 1 WinBUGS codes (PDF 263 kb) [file 12885_2019_6039_MOESM1_ESM.pdf]

Additional file 1:

Table S1 Supplementary characteristics of trials and patients in the 21 eligible RCTs (10187 patients).

Table S2 Risk of bias assessment.

Table S3 Efficacy and tolerability of all treatments according to pairwise estimates.

Table S4 Inconsistency evaluation.

Table S5 Efficacy and tolerability of all treatments according to Bayesian network meta-analysis.

Appendix 1 WinBUGS codes.

Additional file 1

Table S1 Supplementary characteristics of trials and patients in the 21 eligible RCTs (10187 patients).

Author Year	Treatment	Patients (N)	ECOG Performance Status (%)			Outcomes					
			0	1	2	MMR within 12 months (%)	CCyR within 12 months (%)	Overall Discontinuation (%)	Discontinuation for drug-related AEs (%)	Progression to AP-CML (%)	Serious AEs (%)
O'Brien(1) 2003	Imatinib 400mg QD	553	77	21	1	28.8	73.8	12.3	2	6.9	/
	IFN-α + Ara-C	553	74	21	2	0.17	8.5	31.6	6	12.8	/
Cortes(1) 2009	Imatinib 800mg QD	319	76.5	20.1	1.6	46.4	69.9	19.7	10.0	1.9	64.0
	Imatinib 400mg QD	157	79.0	17.8	1.3	40.1	65.6	15.9	4.4	3.2	33.0
Baccarani 2009	Imatinib 800mg QD	108	/	/	/	49	64	/	16.7	/	/
	Imatinib 400mg QD	108	/	/	/	41	58	/	9.3	/	/
Preudhomme 2010	Imatinib 400mg QD	159	/	/	/	38	58	34.6	6.3	/	/
	Imatinib 600mg QD	160	/	/	/	49	65	36.3	10.6	/	/
	Imatinib 400mg QD + Ara-C	158	/	/	/	46	70	93.1	42.1	/	/
	Imatinib 400mg QD + IFN-α	160	/	/	/	57	66	83.1	60.6	/	/
Saglio 2010	Nilotinib 300mg BID	282	/	/	/	55	80	21.6	9.3	3.6	/
	Nilotinib 400mg BID	281	/	/	/	51	78	25.3	12.6	2.2	/
	Imatinib 400mg QD	283	/	/	/	27	65	25.8	10.4	7.5	/

Kantarjian 2010	Dasatinib 100mg QD	259	82	18	0	46	83	15.5	7	10.8	/
	Imatinib 400mg QD	260	79	20	1	28	72	18.6	5	13.8	/
Petzer 2010	Imatinib 800mg QD	113	/	/	/	32.1	61	/	/	/	/
	Imatinib 400mg QD	113	/	/	/	25.4	36	/	/	/	/
Cortes(2) 2011	Bosutinib 500mg QD	250	74	26	0	41	70	28.4	24	5	64
	Imatinib 400mg QD	252	72	28	0	27	68	19.8	7	2	48
Hehlmann (1) 2011	Imatinib 800mg QD	338	37	49	14	54.8	62.9	9.5	/	/	/
	Imatinib 400mg QD	325	35	53	12	30.8	49.4	9.7	/	/	/
	Imatinib 400mg QD + IFN-α	351	34	55	11	34.7	49.7	7.4	/	/	/
Radich 2012	Dasatinib 100mg QD	123	58	39	3	59	84	20	13	/	58
	Imatinib 400mg QD	123	63	36	1	44	69	28	10	/	35
Thielen 2013	Imatinib 400mg QD	55	/	/	/	56	69	17.9	14	/	/
	Imatinib 400mg QD + Ara-C	54	/	/	/	48	80	33.9	18	/	/
Hughes 2014	Nilotinib 400mg BID	104	/	/	/	32.7	/	23.1	11.5	/	14
	Imatinib 400mg QD	103	/	/	/	13.5	/	8.7	2.9	/	11
O’Brien(2) 2014	Dasatinib 100mg QD	407	/	/	/	58.4	53.4	14.5	22.2	/	/
	Imatinib 400mg QD	407	/	/	/	43.1	41.6	19.7	15.5	/	/
Deininger 2014	Imatinib 800mg QD	73	68	31	1	53	85	31	17.8	/	58
	Imatinib 400mg QD	72	61	35	4	36	67	233	8.2	/	31
Hjorth-Han sen 2015	Dasatinib 100mg QD	22	41	54	5	80	100	36.3	32	/	59.1
	Imatinib 400mg QD	24	29	58	13	47	95	29.1	29	/	41.7
Wang	Nilotinib 300mg BID	134	/	/	/	56	80.5	13.4	3	/	68.4

2015	Imatinib 400mg QD	133	/	/	/	30.8	77.6	10.5	1.5	/	59.8
Kwak 2015	Radotinib 300mg BID	79	67	33	0	52	91	12.7	8.9	/	/
	Radotinib 400mg BID	81	68	31	1	46	82	28.4	19.8	/	/
	Imatinib 400mg QD	81	63	36	1	30	77	18.5	6.2	/	/
Lipton 2016	Ponatinib 45mg QD	155	75	24	1	80	/	15.5	9	/	/
	Imatinib 400mg QD	152	78	21	1	38	/	7.2	1.3	/	/
Cortes(3) 2016	Nilotinib 400mg BID	96	81	18	1	42	66	21.6	8.3	7	38
	Imatinib 600mg QD	95	84	15	1	19	40	36.5	3.8	4	31
Hehlmann (2) 2017	Imatinib 400mg QD	400	36	51	13	36.7	67.5	/	/	4.3	14.7
	Imatinib 400mg QD + IFN-α	430	35	53	12	43.1	67.8	/	/	3.6	6.5
	Imatinib 400mg QD + Ara-C	158	35	51	14	29.6	53.6	/	/	5.7	6.3
	Imatinib 400mg QD after IFN-α	128	38	62	1	10	25.7	/	/	5.1	1
	Imatinib 800mg QD	420	38	48	14	55.6	77.6	/	/	4.8	29.5
Cortes(4) 2018	Bosutinib 400mg QD	268	71	29	0	47.2	77.7	22	12.7	0.4	56.3
	Imatinib 400mg QD	268	71	29	0.5	36.9	66.4	26.8	8.7	1.5	42.6

(“/” means “not available”)

Table S2 Risk of bias assessment.

Source	Adequate sequence generation?	Allocation concealment?	Blinding? (Self-reported outcome)	Blinding? (Objective outcomes)	Incomplete outcome data addressed?	Free of selective reporting?	Other bias?
O'Brien(1) 2003	Yes	Yes	Yes	Yes	Yes	Yes	Unclear
Cortes(1) 2009	Yes	Yes	Yes	Yes	Yes	Yes	Unclear
Baccarani 2009	Yes	Yes	Yes	Yes	Yes	Yes	Unclear
Preudhomme 2010	Yes	Yes	No	Yes	Yes	Yes	Unclear
Saglio 2010	Yes	Yes	Yes	Yes	Yes	Yes	Unclear
Kantarjian 2010	Yes	Yes	Yes	Yes	Yes	Yes	Unclear
Petzer 2010	Yes	Unclear	Unclear	Yes	Unclear	Yes	Unclear
Cortes(2) 2011	Yes	Yes	Yes	Yes	Yes	Yes	Unclear
Hehlmann(1) 2011	Yes	Yes	Unclear	Yes	Yes	Yes	Unclear
Radich 2012	Yes	Yes	Yes	Yes	Unclear	Yes	Unclear
Thielen 2013	Yes	Unclear	Yes	Yes	Yes	Yes	Unclear
Hughes(2) 2014	Yes	Yes	Yes	Yes	Yes	Yes	Unclear
O'Brien(2) 2014	Yes	Yes	Yes	Yes	Unclear	Yes	Unclear
Deininger 2014	Yes	Yes	Yes	Yes	Yes	Yes	Unclear
Hjorth-Hansen 2015	Yes	Yes	Unclear	Yes	Yes	Yes	Unclear
Wang 2015	Yes	Yes	Yes	Yes	Yes	Yes	Unclear
Kwak 2015	Yes	Yes	Yes	Yes	Yes	Yes	Unclear
Lipton 2016	Yes	Yes	Yes	Yes	Yes	Yes	Unclear
Cortes(3) 2016	Yes	Yes	Unclear	Yes	Unclear	Yes	Unclear
Hehlmann(2) 2017	No	No	Unclear	Unclear	Unclear	Yes	Unclear
Cortes(4) 2018	Yes	Yes	Yes	Yes	Unclear	Yes	Unclear

Table S3 Efficacy and tolerability of all treatments according to pairwise estimates

Treatment	Studies (N)	Patients (N)	Efficacy OR (95% CI)			Tolerability OR (95% CI)		
			MMR within 12 months	CCyR within 12 months	Progression to AP	Overall discontinuation	Discontinuation for drug-related AEs	Serious AEs
**vs Imatinib 400mg QD**								
IFN-α + Ara-C	1	1106	0.00(0.00, 0.03)	0.03(0.02, 0.05)	2.00(1.32, 3.02)	3.30(2.42, 4.51)	3.13(1.56, 6.26)	-
Imatinib 600mg QD	1	319	1.57(1.01, 2.45)	1.35(0.86, 2.12)	-	1.08(0.68, 1.70)	1.77(0.79, 4.00)	-
Imatinib 800mg QD	6	2546	1.82(1.39, 2.38)	1.60(1.21, 2.12)	0.97(0.54, 1.73)	1.00(0.41, 2.46)	2.83(1.49, 5.36)	1.24(0.18, 8.38)
Imatinib 400mg QD + Ara-C	3	984	0.93(0.58, 1.50)	1.14(0.49, 2.69)	1.13(0.58, 1.73)	5.95(0.33, 106.31)	3.95(0.50,31.26)	0.39(0.19, 0.78)
Imatinib 400mg QD + IFN-α	3	1805	1.44(1.06, 1.97)	1.07(0.89, 1.30)	1.36(0.59, 3.12)	2.61(0.22, 31.61)	22.94(11.23, 46.87)	0.40(0.25, 0.65)
Imatinib 400mg QD after IFN-α	1	528	0.20(0.11, 0.36)	0.17(0.11, 0.26)	0.81(0.40, 1.65)	-	-	0.12(0.01, 0.92)
Nilotinib 300mg BID	2	832	1.24(1.084, 1.429)	1.16(1.07, 1.26)	0.48(0.23, 0.99)	0.92(0.59, 1.45)	0.96(0.57, 1.63)	1.45(0.88, 2.40)
Nilotinib 400mg BID	2	769	1.68(0.56, 5.07)	1.90(1.31, 2.76)	1.30(0.53, 3.22)	1.64(0.53, 5.15)	2.01(0.61, 6.65)	1.27(0.98, 1.63)
Dasatinib 100mg QD	4	1625	1.44(1.29, 1.60)	1.34(1.25, 1.44)	0.78(0.49, 1.24)	0.74(0.58, 0.96)	1.48(1.11, 1.98)	1.54(1.02, 2.31)
Bosutinib 400mg QD	1	502	1.51(1.07, 2.14)	1.75(1.20, 2.57)	0.25(0.03, 2.23)	0.77(0.52, 1.14)	1.55(0.89, 2.71)	1.74(1.24, 2.45)
Bosutinib 500mg QD	1	536	1.90(1.30, 2.76)	1.11(0.76, 1.61)	2.71(0.95, 7.72)	1.60(1.06, 2.42)	4.11(2.34, 7.19)	1.92(1.35, 2.75)
Radotinib 300mg BID	1	160	2.56(1.34, 4.91)	3.15(1.24, 8.00)	-	0.64(0.27, 1.52)	0.14(0.01, 2.81)	-
Radotinib 400mg BID	1	162	2.00(1.05, 3.81)	1.35(0.63, 2.89)	-	1.75(0.83, 3.66)	2.08(0.50, 8.62)	-
Ponatinib 45mg QD	1	307	6.48(3.89, 10.82)	-	-	2.35(1.11, 4.98)	7.50(1.67, 33.60)	-
**vs Imatinib 600mg QD**								
Nilotinib 400mg BID	1	191	3.06(1.59, 5.88)	2.86(1.59, 5.16)	1.79(0.51, 6.33)	0.48(0.25,0.91)	0.09(0.14, 1.66)	1.37(0.75, 2.49)
Imatinib 400mg QD + Ara-C	1	318	0.90(0.58, 1.40)	1.27(0.79, 2.04)	-	23.50(11.76,46.96)	0.74(0.09, 0.29)	-

Imatinib 400mg QD + IFN-α	1	320	1.39(0.89, 2.15)	1.06(0.67, 1.68)	-	8.66(5.13, 14.64)	1.54(0.04, 0.14)	-
**vs Imatinib 800mg QD**								
Imatinib 400mg QD + Ara-C	1	578	0.34(0.23, 0.50)	0.34(0.23, 0.49)	1.21(0.54, 2.71)	-	-	2.30(0.97, 5.43)
Imatinib 400 mg QD + IFN-α	2	1188	0.52(0.38,0.70)	0.59(0.48, 0.74)	0.72(0.36, 1.43)	0.76(0.45, 1.31)	-	2.37(1.19, 4.72)
Imatinib 400 mg QD after IFN-α	1	548	0.09(0.05, 0.16)	0.10(0.06, 0.16)	1.16(0.48, 2.80)	-	-	0.27(0.03, 2.08)
**vs Imatinib 400mg QD + Ara-C**								
Imatinib 400mg QD + IFN-α	1	588	1.78(1.21, 2.64)	1.82(1.25, 2.64)	0.60(0.26, 1.40)	0.37(0.18, 0.77)	1.54(0.31, 0.75)	1.03(0.49, 2.17)
Imatinib 400mg QD after IFN-α	1	286	0.27(0.14, 0.52)	0.30(0.18, 0.49)	0.96(0.35, 2.65)	-	-	0.12(0.01, 0.92)
**vs Imatinib 400mg QD + IFN-α**								
Imatinib 400mg QD after IFN-α	1	558	0.15(0.08, 0.27)	1.00(0.57, 1.75)	1.60(0.64, 4.02)	-	-	0.11(0.02, 0.84)
**vs Nilotinib 300mg BID**								
Nilotinib 400mg BID	1	563	0.85(0.61, 1.18)	0.88(0.58, 1.31)	0.59(0.21, 1.66)	1.22(0.83, 1.81)	1.40(0.82, 2.40)	-
**vs Radotinib 300mg BID**								
Radotinib 400mg BID	1	160	0.78(0.42, 1.45)	0.43(0.16, 1.11)	-	2.74(1.20, 6.22)	0.25(0.22, 0.73)	-

("-" means "cannot be estimated for lack of primary data"

Table S4 Inconsistency evaluation

Inconsistency evaluation was estimated by the **node-splitting method** which generates P values for the null hypothesis that there is no significant inconsistency between direct and indirect evidence. "P > 0.05" and "Inconsistency factors include 0" means no significant inconsistency.

"A" to "O" refer to each treatment. (A: Imatinib 400mg QD; B: Imatinib 600mg QD; C: Imatinib 800mg QD; D: IFN-α + Ara-C; E: Imatinib 400mg QD + Ara-C; F: Imatinib 400mg QD + IFN-α; G: Nilotinib 300mg BID; H: Nilotinib 400mg BID; I: Dasatinib 100mg QD; J: Radotinib 300mg BID; K: Radotinib 400mg BID; L: Bosutinib 400mg QD; M: Bosutinib 500mg QD; N: Ponatinib 45mg QD; O: Imatinib 400mg QD after IFN-α.)

(A) MMR within 12 months

Name	Direct Effect	Indirect Effect	Overall	P-Value
A, B	0.24 (-0.37, 0.97)	-0.08 (-1.05, 0.86)	0.05 (-0.44, 0.51)	0.57
A, H	1.05 (0.58, 1.56)	1.21 (0.27, 2.23)	1.08 (0.67, 1.50)	0.78
B, E	-0.07 (-0.70, 0.52)	-0.47 (-1.12, 0.18)	-0.24 (-0.76, 0.28)	0.31
B, F	0.38 (-0.27, 0.97)	0.02 (-0.56, 0.59)	0.18 (-0.32, 0.69)	0.33
B, H	1.13 (0.35, 1.98)	0.95 (0.23, 1.70)	1.03 (0.51, 1.59)	0.71
C, E	-1.08 (-1.70, -0.44)	-0.72 (-1.23, -0.16)	-0.86 (-1.27, -0.46)	0.34
C, F	-0.59 (-1.00, -0.25)	-0.15 (-0.73, 0.46)	-0.45 (-0.76, -0.08)	0.16
E, F	0.50 (0.09, 0.93)	0.14 (-0.46, 0.74)	0.41 (0.03, 0.83)	0.24
G, H	-0.16 (-0.73, 0.46)	0.01 (-0.77, 0.83)	-0.10 (-0.57, 0.40)	0.69

Cycle	Inconsistency factor Median (95% CI)
A, B, H	0.00 (-0.53, 0.58)
A, B, G, H	0.05 (-0.41, 0.63)
A, C, E	-0.09 (-0.82, 0.43)
A, C, E	-0.09 (-0.82, 0.43)
A, C, E, F	-0.10 (-0.75, 0.47)
A, C, E, F	-0.10 (-0.75, 0.47)
C, E, F	0.15 (-0.20, 0.61)

(b) CCyR within 12 months

Name	Direct Effect	Indirect Effect	Overall	P-Value
A, B	0.19 (-0.61, 1.07)	-0.53 (-1.70, 0.68)	-0.11 (-0.71, 0.50)	0.28
A, H	0.62 (-0.17, 1.44)	1.11 (-0.02, 2.26)	0.71 (0.09, 1.30)	0.45
B, E	0.31 (-0.38, 0.96)	-0.32 (-0.98, 0.45)	0.07 (-0.57, 0.75)	0.17
B, F	0.09 (-0.78, 0.93)	0.25 (-0.56, 1.07)	0.21 (-0.43, 0.85)	0.75
B, H	1.08 (0.18, 2.00)	0.50 (-0.59, 1.54)	0.81 (0.15, 1.47)	0.37
C, E	-1.07 (-1.65, -0.53)	-0.13 (-0.67, 0.36)	-0.64 (-1.13, -0.12)	0.02
C, F	-0.51 (-1.06, 0.04)	-0.59 (-1.39, 0.23)	-0.51 (-0.94, -0.07)	0.88
E, F	0.22 (-0.40, 0.85)	0.05 (-0.90, 0.92)	0.13 (-0.38, 0.62)	0.70

G, H	-0.08 (-0.88, 0.67)	0.59 (-0.71, 1.88)	0.09 (-0.57, 0.79)	0.33

Cycle	Inconsistency factor Median (95% CI)
A, B, H	-0.00 (-0.53, 0.58)
A, B, G, H	0.03 (-0.33, 0.95)
A, C, E	-0.01 (-0.76, 0.47)
A, C, E	-0.01 (-0.76, 0.47)
A, C, E, F	0.00 (-0.57, 0.63)
A, C, E, F	0.00 (-0.57, 0.63)
C, E, F	-0.00 (-0.44, 0.51)

(c) Progression to AP-CML: No cycle

(d) Serious AEs: No cycle

(e) Overall discontinuation

Name	Direct Effect	Indirect Effect	Overall	P-Value
A, B	-0.02 (-1.92, 2.02)	1.28 (-1.08, 3.74)	-0.13 (-1.68, 1.37)	0.36
A, H	0.52 (-0.91, 1.96)	-1.59 (-4.28, 1.08)	0.10 (-1.23, 1.40)	0.14
B, E	3.16 (1.16, 5.26)	0.73 (-1.94, 3.27)	2.14 (0.34, 3.98)	0.12
B, F	2.12 (0.46, 3.78)	-0.39 (-2.33, 1.66)	1.03 (-0.71, 2.79)	0.05
B, H	-0.74 (-2.78, 1.29)	1.44 (-0.77, 3.74)	0.24 (-1.37, 1.84)	0.13
C, F	-0.29 (-1.96, 1.44)	1.83 (-0.06, 3.64)	0.48 (-1.16, 2.05)	0.09
E, F	-0.81 (-2.64, 0.89)	-2.57 (-4.47, -0.53)	-1.10 (-2.89, 0.65)	0.17
G, H	0.20 (-2.01, 2.47)	0.06 (-2.45, 2.59)	0.09 (-1.57, 1.76)	0.92

Cycle	Inconsistency factor Median (95% CI)
A, C, F	-0.02 (-2.21, 2.09)
A, C, F	-0.02 (-2.21, 2.09)
A, B, C, F, H	-0.48 (-3.31, 0.84)
A, B, C, F, G, H	-0.10 (-2.37, 1.32)
A, C, E, F	0.45 (-0.82, 2.99)

(f) Discontinuation for drug-related AEs

Name	Direct Effect	Indirect Effect	Overall	P-Value
A, B	0.30 (-1.03, 1.93)	-0.16 (-2.11, 1.99)	0.03 (-1.00, 1.18)	0.68
A, H	0.62 (-0.30, 1.85)	0.99 (-1.37, 3.06)	0.70 (-0.06, 1.70)	0.75
B, E	1.88 (1.11, 2.77)	-0.11 (-1.42, 1.45)	1.57 (0.24, 2.58)	0.02
B, H	0.80 (-0.96, 2.65)	0.61 (-1.00, 2.51)	0.66 (-0.43, 1.91)	0.89
G, H	0.29 (-0.92, 1.50)	1.20 (-0.53, 2.83)	0.45 (-0.62, 1.59)	0.31

Cycle	Inconsistency factor Median (95% CI)
A, B, G, H	0.95 (-0.54, 2.24)
A, B, E, G, H	-0.71 (-2.34, 0.62)
A, G, H	-0.90 (-2.49, 0.24)

Table S5 Efficacy and tolerability of all treatments according to Bayesian network meta-analysis

(a) MMR within 12 months

Imatinib 400mg QD	1.05 (0.65, 1.67)	1.96 (1.49, 2.48)	0.00 (0.00, 0.02)	0.83 (0.56, 1.19)	1.25 (0.91, 1.72)	3.24 (2.10, 4.95)	2.95 (1.95, 4.47)	2.06 (1.51, 2.97)	2.59 (1.16, 5.75)	2.01 (0.92, 4.49)	1.51 (0.85, 2.73)	1.88 (1.03, 3.47)	6.67 (3.30, 13.29)	0.18 (0.09, 0.37)
0.96 (0.60, 1.55)	Imatinib 600mg QD	1.87 (1.10, 3.12)	0.00 (0.00, 0.02)	0.79 (0.47, 1.32)	1.19 (0.72, 2.00)	3.08 (1.67, 5.74)	2.81 (1.66, 4.92)	1.98 (1.14, 3.63)	2.44 (0.99, 6.35)	1.91 (0.78, 4.91)	1.44 (0.70, 3.14)	1.80 (0.85, 3.92)	6.23 (2.76, 14.84)	0.18 (0.07, 0.40)
0.51 (0.40, 0.67)	0.54 (0.32, 0.91)	Imatinib 800mg QD	0.00 (0.00, 0.01)	0.42 (0.28, 0.63)	0.64 (0.47, 0.92)	1.66 (1.01, 2.77)	1.50 (0.94, 2.50)	1.05 (0.72, 1.69)	1.32 (0.58, 3.11)	1.02 (0.46, 2.43)	0.77 (0.42, 1.51)	0.96 (0.51, 1.90)	3.38 (1.63, 7.13)	0.09 (0.04, 0.19)
302.56 (51.19, 4111.48)	313.50 (49.36, 4377.39)	588.80 (99.19, 7836.26)	IFN + Ara-C	249.74 (40.67, 3429.57)	380.31 (62.20, 5265.31)	981.73 (156.86, 13641.65)	896.95 (143.78, 12260.57)	632.83 (103.57, 8822.02)	793.41 (106.17, 12296.72)	613.89 (84.50, 9426.49)	466.09 (70.27, 6540.84)	578.18 (87.08, 8398.08)	2007.28 (294.80, 30945.78)	55.26 (7.89, 795.17)
1.21 (0.84, 1.77)	1.27 (0.76, 2.15)	2.36 (1.58, 3.54)	0.00 (0.00, 0.02)	Imatinib 400mg QD + Ara-C	1.51 (1.03, 2.30)	3.90 (2.25, 6.94)	3.56 (2.09, 6.24)	2.51 (1.54, 4.32)	3.10 (1.31, 7.75)	2.43 (1.02, 5.94)	1.83 (0.93, 3.72)	2.28 (1.16, 4.72)	7.98 (3.67, 17.70)	0.22 (0.10, 0.47)
0.80 (0.58, 1.10)	0.84 (0.50, 1.38)	1.56 (1.09, 2.14)	0.00 (0.00, 0.02)	0.66 (0.43, 0.97)	Imatinib 400mg QD + IFN	2.58 (1.51, 4.35)	2.34 (1.41, 3.87)	1.65 (1.06, 2.65)	2.03 (0.87, 4.91)	1.60 (0.69, 3.77)	1.21 (0.62, 2.36)	1.50 (0.77, 2.99)	5.23 (2.46, 11.34)	0.14 (0.07, 0.29)
0.31 (0.20, 0.48)	0.32 (0.17, 0.60)	0.60 (0.36, 0.99)	0.00 (0.00, 0.01)	0.26 (0.14, 0.44)	0.39 (0.23, 0.66)	Nilotinib 300mg BID	0.91 (0.57, 1.50)	0.64 (0.38, 1.14)	0.79 (0.33, 1.99)	0.62 (0.26, 1.55)	0.47 (0.23, 0.98)	0.58 (0.28, 1.25)	2.03 (0.91, 4.69)	0.06 (0.02, 0.13)
0.34 (0.22, 0.51)	0.36 (0.20, 0.60)	0.67 (0.40, 1.06)	0.00 (0.00, 0.01)	0.28 (0.16, 0.48)	0.43 (0.26, 0.71)	1.10 (0.67, 1.77)	Nilotinib 400mg BID	0.70 (0.42, 1.21)	0.89 (0.36, 2.15)	0.68 (0.28, 1.68)	0.51 (0.25, 1.06)	0.64 (0.31, 1.33)	2.26 (1.01, 4.98)	0.06 (0.03, 0.14)
0.48 (0.34, 0.66)	0.51 (0.28, 0.88)	0.95 (0.59, 1.39)	0.00 (0.00, 0.01)	0.40 (0.23, 0.65)	0.61 (0.38, 0.94)	1.56 (0.88, 2.65)	1.43 (0.82, 2.40)	Dasatinib 100mg QD	1.25 (0.52, 2.96)	0.97 (0.40, 2.25)	0.74 (0.36, 1.42)	0.91 (0.45, 1.78)	3.22 (1.45, 6.82)	0.09 (0.04, 0.19)
0.39 (0.17, 0.86)	0.41 (0.16, 1.01)	0.76 (0.32, 1.73)	0.00 (0.00, 0.01)	0.32 (0.13, 0.76)	0.49 (0.20, 1.15)	1.27 (0.50, 3.05)	1.13 (0.46, 2.78)	0.80 (0.34, 1.93)	Radotinib 300mg BID	0.78 (0.35, 1.68)	0.58 (0.22, 1.59)	0.72 (0.27, 1.98)	2.58 (0.87, 7.30)	0.07 (0.02, 0.21)
0.50 (0.22, 1.09)	0.52 (0.20, 1.29)	0.98 (0.41, 2.19)	0.00 (0.00, 0.01)	0.41 (0.17, 0.98)	0.62 (0.27, 1.45)	1.60 (0.64, 3.92)	1.47 (0.59, 3.57)	1.04 (0.44, 2.47)	1.28 (0.59, 2.83)	Radotinib 300mg BID	0.75 (0.28, 2.03)	0.94 (0.35, 2.55)	3.27 (1.13, 9.55)	0.09 (0.03, 0.27)
0.66 (0.37, 1.18)	0.70 (0.32, 1.43)	1.30 (0.66, 2.37)	0.00 (0.00, 0.01)	0.55 (0.27, 1.07)	0.83 (0.42, 1.62)	2.14 (1.02, 4.41)	1.96 (0.94, 4.00)	1.36 (0.70, 2.75)	1.72 (0.63, 4.60)	1.33 (0.49, 3.56)	Bosutinib 400mg QD	1.24 (0.54, 2.89)	4.40 (1.75, 10.79)	0.12 (0.05, 0.30)
0.53 (0.29, 0.97)	0.55 (0.26, 1.18)	1.04 (0.53, 1.95)	0.00 (0.00, 0.01)	0.44 (0.21, 0.86)	0.67 (0.33, 1.30)	1.72 (0.80, 3.59)	1.57 (0.75, 3.21)	1.10 (0.56, 2.22)	1.38 (0.50, 3.74)	1.06 (0.39, 2.86)	0.81 (0.35, 1.86)	Bosutinib 500mg QD	3.54 (1.40, 8.63)	0.10 (0.04, 0.25)
0.15 (0.08, 0.30)	0.16 (0.07, 0.36)	0.30 (0.14, 0.61)	0.00 (0.00, 0.00)	0.13 (0.06, 0.27)	0.19 (0.09, 0.41)	0.49 (0.21, 1.10)	0.44 (0.20, 0.99)	0.31 (0.15, 0.69)	0.39 (0.14, 1.15)	0.31 (0.10, 0.89)	0.23 (0.09, 0.57)	0.28 (0.12, 0.71)	Ponatinib 45mg QD	0.03 (0.01, 0.08)
5.53 (2.71, 11.38)	5.71 (2.52, 13.67)	10.72 (5.22, 22.54)	0.02 (0.00, 0.13)	4.51 (2.14, 9.86)	6.90 (3.39, 14.60)	17.81 (7.78, 41.68)	16.22 (7.15, 37.66)	11.45 (5.26, 25.82)	14.51 (4.85, 42.10)	11.07 (3.77, 32.65)	8.42 (3.31, 21.17)	10.46 (4.07, 26.28)	36.33 (13.24, 100.98)	Imatinib 400mg QD after IFN-α

(b) CCyR within 12 months

Imatinib 400mg QD	0.90 (0.49, 1.65)	1.83 (1.35, 2.57)	0.03 (0.02, 0.07)	0.97 (0.63, 1.54)	1.10 (0.76, 1.67)	1.84 (1.01, 3.21)	2.03 (1.09, 3.68)	1.89 (1.23, 3.05)	3.37 (1.09, 11.18)	1.39 (0.51, 3.76)	1.77 (0.81, 3.78)	1.09 (0.50, 2.38)	0.20 (0.10, 0.39)
1.12 (0.61, 2.03)	Imatinib 600mg QD	2.03 (1.07, 3.99)	0.04 (0.01, 0.10)	1.08 (0.57, 2.11)	1.23 (0.65, 2.34)	2.03 (0.92, 4.39)	2.25 (1.17, 4.34)	2.09 (1.00, 4.54)	3.76 (1.02, 14.11)	1.54 (0.47, 4.96)	1.97 (0.74, 5.11)	1.22 (0.47, 3.26)	0.22 (0.09, 0.52)
0.55 (0.39, 0.74)	0.49 (0.25, 0.94)	Imatinib 800mg QD	0.02 (0.01, 0.04)	0.53 (0.32, 0.88)	0.60 (0.39, 0.93)	1.00 (0.51, 1.85)	1.10 (0.55, 2.13)	1.03 (0.60, 1.81)	1.84 (0.57, 6.31)	0.76 (0.26, 2.15)	0.96 (0.41, 2.17)	0.60 (0.25, 1.38)	0.11 (0.05, 0.21)
30.77 (14.13, 64.45)	27.83 (10.47, 70.97)	56.07 (24.89, 127.31)	IFN + Ara-C	29.91 (12.65, 72.38)	33.95 (14.63, 79.40)	56.67 (21.15, 142.01)	62.06 (23.33, 158.48)	57.97 (24.23, 144.77)	104.66 (26.59, 403.52)	42.95 (12.14, 148.27)	54.58 (18.19, 154.56)	33.58 (11.52, 97.34)	5.97 (2.16, 16.57)
1.03 (0.65, 1.59)	0.93 (0.47, 1.77)	1.89 (1.13, 3.11)	0.03 (0.01, 0.08)	Imatinib 400mg QD + Ara-C	1.14 (0.68, 1.86)	1.88 (0.89, 3.77)	2.08 (0.99, 4.15)	1.94 (1.05, 3.68)	3.45 (1.04, 12.28)	1.43 (0.47, 4.26)	1.82 (0.73, 4.30)	1.12 (0.45, 2.75)	0.20 (0.09, 0.41)
0.91 (0.60, 1.32)	0.81 (0.43, 1.53)	1.66 (1.08, 2.57)	0.03 (0.01, 0.07)	0.88 (0.54, 1.47)	Imatinib 400mg QD + IFN	1.66 (0.81, 3.23)	1.83 (0.91, 3.66)	1.70 (0.94, 3.18)	3.08 (0.91, 10.70)	1.26 (0.42, 3.69)	1.60 (0.66, 3.72)	0.99 (0.41, 2.36)	0.18 (0.08, 0.35)
0.54 (0.31, 0.99)	0.49 (0.23, 1.08)	1.00 (0.54, 1.98)	0.02 (0.01, 0.05)	0.53 (0.27, 1.12)	0.60 (0.31, 1.23)	Nilotinib 300mg BID	1.10 (0.56, 2.20)	1.03 (0.52, 2.22)	1.86 (0.53, 6.94)	0.76 (0.24, 2.42)	0.97 (0.38, 2.49)	0.60 (0.23, 1.59)	0.11 (0.04, 0.27)
0.49 (0.27, 0.91)	0.44 (0.23, 0.86)	0.91 (0.47, 1.83)	0.02 (0.01, 0.04)	0.48 (0.24, 1.01)	0.55 (0.27, 1.10)	0.91 (0.45, 1.77)	Nilotinib 400mg BID	0.93 (0.45, 2.03)	1.67 (0.47, 6.32)	0.69 (0.21, 2.24)	0.87 (0.32, 2.32)	0.54 (0.20, 1.45)	0.10 (0.04, 0.24)
0.53 (0.33, 0.81)	0.48 (0.22, 1.00)	0.97 (0.55, 1.66)	0.02 (0.01, 0.04)	0.51 (0.27, 0.95)	0.59 (0.31, 1.06)	0.97 (0.45, 1.92)	1.07 (0.49, 2.21)	Dasatinib 100mg QD	1.78 (0.51, 6.29)	0.74 (0.24, 2.19)	0.93 (0.37, 2.25)	0.58 (0.24, 1.39)	0.10 (0.04, 0.23)
0.30 (0.09, 0.92)	0.27 (0.07, 0.98)	0.54 (0.16, 1.75)	0.01 (0.00, 0.04)	0.29 (0.08, 0.96)	0.32 (0.09, 1.10)	0.54 (0.14, 1.87)	0.60 (0.16, 2.15)	0.56 (0.16, 1.94)	Radotinib 300mg BID	0.41 (0.12, 1.28)	0.52 (0.13, 2.00)	0.33 (0.08, 1.27)	0.06 (0.01, 0.22)
0.72 (0.27, 1.96)	0.65 (0.20, 2.11)	1.32 (0.47, 3.78)	0.02 (0.01, 0.08)	0.70 (0.23, 2.14)	0.79 (0.27, 2.37)	1.32 (0.41, 4.19)	1.46 (0.45, 4.68)	1.36 (0.46, 4.15)	2.46 (0.78, 8.30)	Radotinib 400mg BID	1.26 (0.37, 4.47)	0.79 (0.23, 2.81)	0.14 (0.04, 0.47)
0.57 (0.26, 1.24)	0.51 (0.20, 1.34)	1.04 (0.46, 2.44)	0.02 (0.01, 0.05)	0.55 (0.23, 1.38)	0.62 (0.27, 1.51)	1.04 (0.40, 2.66)	1.15 (0.43, 3.10)	1.07 (0.44, 2.73)	1.93 (0.50, 7.79)	0.79 (0.22, 2.71)	Bosutinib 400mg QD	0.62 (0.21, 1.87)	0.11 (0.04, 0.31)
0.92 (0.42, 2.02)	0.82 (0.31, 2.15)	1.68 (0.73, 3.97)	0.03 (0.01, 0.09)	0.89 (0.36, 2.22)	1.01 (0.42, 2.46)	1.67 (0.63, 4.30)	1.84 (0.69, 4.97)	1.73 (0.72, 4.25)	3.05 (0.78, 12.56)	1.26 (0.36, 4.44)	1.61 (0.53, 4.77)	Bosutinib 500mg QD	0.18 (0.06, 0.49)
5.12 (2.58, 10.33)	4.62 (1.91, 11.32)	9.41 (4.67, 19.54)	0.17 (0.06, 0.46)	4.99 (2.41, 10.79)	5.68 (2.82, 11.80)	9.46 (3.77, 22.60)	10.34 (4.23, 25.73)	9.68 (4.36, 22.78)	17.57 (4.61, 68.96)	7.19 (2.11, 24.26)	9.09 (3.22, 25.82)	5.67 (2.04, 16.04)	Imatinib 400mg QD after IFN-α

(c) Progression to AP-CML

Imatinib 400mg QD	0.14 (0.01, 1.64)	0.93 (0.27, 2.78)	2.00 (0.44, 8.68)	1.23 (0.26, 5.54)	0.75 (0.15, 3.13)	0.44 (0.09, 2.42)	0.26 (0.05, 1.35)	0.77 (0.16, 3.17)	0.20 (0.00, 2.04)	2.86 (0.55, 17.08)	1.13 (0.22, 5.18)
7.04 (0.61, 97.68)	Imatinib 600mg QD	6.30 (0.40, 105.19)	14.34 (0.77, 276.62)	8.58 (0.47, 161.87)	5.17 (0.28, 98.46)	3.11 (0.25, 43.63)	1.83 (0.30, 13.08)	5.16 (0.28, 108.92)	1.27 (0.02, 44.41)	19.82 (0.99, 447.41)	7.82 (0.40, 156.14)
1.08 (0.36, 3.77)	0.16 (0.01, 2.50)	Imatinib 800mg QD	2.13 (0.35, 15.36)	1.27 (0.30, 6.40)	0.79 (0.18, 3.55)	0.47 (0.07, 4.20)	0.27 (0.04, 2.37)	0.79 (0.13, 5.84)	0.22 (0.00, 3.08)	3.14 (0.41, 27.58)	1.19 (0.27, 6.15)
0.50 (0.12, 2.25)	0.07 (0.00, 1.29)	0.47 (0.07, 2.83)	IFN + Ara-C	0.61 (0.07, 5.02)	0.37 (0.04, 2.90)	0.22 (0.03, 2.05)	0.13 (0.01, 1.26)	0.38 (0.04, 3.19)	0.10 (0.00, 1.58)	1.44 (0.16, 14.59)	0.57 (0.07, 4.97)
0.81 (0.18, 3.92)	0.12 (0.01, 2.13)	0.79 (0.16, 3.37)	1.63 (0.20, 14.31)	Imatinib 400mg QD + Ara-C	0.61 (0.12, 3.12)	0.37 (0.04, 3.79)	0.22 (0.02, 2.18)	0.62 (0.07, 5.54)	0.17 (0.00, 2.94)	2.41 (0.25, 25.68)	0.94 (0.17, 5.16)
1.34 (0.32, 6.52)	0.19 (0.01, 3.60)	1.27 (0.28, 5.70)	2.68 (0.34, 23.39)	1.63 (0.32, 8.60)	Imatinib 400mg QD + IFN	0.58 (0.07, 6.15)	0.34 (0.04, 3.61)	0.99 (0.12, 9.37)	0.28 (0.01, 4.37)	3.87 (0.43, 42.11)	1.49 (0.29, 8.35)
2.29 (0.41, 11.22)	0.32 (0.02, 3.98)	2.15 (0.24, 13.80)	4.61 (0.49, 39.87)	2.70 (0.26, 25.09)	1.72 (0.16, 14.37)	Nilotinib 300mg BID	0.58 (0.10, 3.23)	1.71 (0.18, 14.69)	0.46 (0.01, 6.99)	6.46 (0.61, 72.33)	2.52 (0.25, 23.41)
3.80 (0.74, 21.47)	0.55 (0.08, 3.38)	3.70 (0.42, 26.17)	7.80 (0.79, 70.07)	4.63 (0.46, 47.14)	2.97 (0.28, 26.48)	1.73 (0.31, 10.14)	Nilotinib 400mg BID	2.95 (0.30, 26.83)	0.78 (0.01, 13.94)	11.08 (0.97, 127.61)	4.24 (0.42, 42.86)
1.30 (0.32, 6.07)	0.19 (0.01, 3.54)	1.26 (0.17, 7.91)	2.65 (0.31, 23.37)	1.60 (0.18, 13.64)	1.01 (0.11, 8.21)	0.59 (0.07, 5.41)	0.34 (0.04, 3.37)	Dasatinib 100mg QD	0.28 (0.01, 4.01)	3.86 (0.39, 39.06)	1.50 (0.16, 13.54)
4.93 (0.49, 202.49)	0.79 (0.02, 57.89)	4.54 (0.32, 202.43)	10.04 (0.63, 501.16)	5.98 (0.34, 301.55)	3.60 (0.23, 170.09)	2.18 (0.14, 124.83)	1.29 (0.07, 72.78)	3.63 (0.25, 192.10)	Bosutinib 400mg QD	14.54 (0.72, 851.44)	5.60 (0.34, 293.59)
0.35 (0.06, 1.82)	0.05 (0.00, 1.01)	0.32 (0.04, 2.46)	0.70 (0.07, 6.36)	0.41 (0.04, 3.98)	0.26 (0.02, 2.31)	0.15 (0.01, 1.63)	0.09 (0.01, 1.03)	0.26 (0.03, 2.53)	0.07 (0.00, 1.38)	Bosutinib 500mg QD	0.39 (0.04, 3.87)
0.88 (0.19, 4.53)	0.13 (0.01, 2.47)	0.84 (0.16, 3.69)	1.77 (0.20, 15.35)	1.07 (0.19, 6.00)	0.67 (0.12, 3.43)	0.40 (0.04, 4.00)	0.24 (0.02, 2.39)	0.67 (0.07, 6.18)	0.18 (0.00, 2.98)	2.57 (0.26, 27.75)	Ponatinib 45mg QD

(d) Serious AEs

Imatinib 400mg QD	1.05 (0.01, 140.67)	1.22 (0.15, 9.09)	0.98 (0.04, 26.61)	1.03 (0.04, 27.99)	1.47 (0.04, 46.10)	1.42 (0.04, 53.72)	2.31 (0.17, 26.85)	1.68 (0.05, 64.95)	1.93 (0.06, 65.07)	0.07 (0.00, 2.92)
0.95 (0.01, 168.63)	Imatinib 600mg QD	1.18 (0.00, 308.38)	0.93 (0.00, 388.44)	0.98 (0.00, 472.16)	1.37 (0.00, 676.46)	1.38 (0.04, 51.24)	2.17 (0.01, 632.42)	1.56 (0.00, 1135.62)	1.85 (0.00, 803.69)	0.07 (0.00, 38.24)
0.82 (0.11, 6.61)	0.85 (0.00, 224.67)	Imatinib 800mg QD	0.79 (0.03, 21.90)	0.84 (0.03, 24.59)	1.19 (0.02, 73.30)	1.16 (0.02, 77.27)	1.88 (0.07, 48.85)	1.38 (0.03, 87.33)	1.63 (0.03, 91.20)	0.06 (0.00, 2.55)
1.02 (0.04, 28.15)	1.08 (0.00, 444.53)	1.26 (0.05, 32.77)	Imatinib 400mg QD + Ara-C	1.07 (0.03, 37.93)	1.51 (0.01, 172.63)	1.47 (0.01, 181.33)	2.31 (0.03, 133.46)	1.71 (0.01, 230.79)	1.93 (0.02, 263.54)	0.07 (0.00, 4.23)
0.97 (0.04, 25.33)	1.02 (0.00, 427.72)	1.20 (0.04, 32.28)	0.94 (0.03, 33.68)	Imatinib 400mg QD + IFN	1.40 (0.01, 168.76)	1.36 (0.01, 165.31)	2.14 (0.03, 140.34)	1.59 (0.01, 201.84)	1.90 (0.02, 222.33)	0.07 (0.00, 3.98)
0.68 (0.02, 23.76)	0.73 (0.00, 365.65)	0.84 (0.01, 45.37)	0.66 (0.01, 86.03)	0.72 (0.01, 92.23)	Nilotinib 300mg BID	0.97 (0.01, 158.26)	1.56 (0.02, 117.92)	1.17 (0.01, 178.92)	1.35 (0.01, 190.56)	0.05 (0.00, 8.02)
0.70 (0.02, 25.48)	0.72 (0.02, 26.44)	0.86 (0.01, 56.64)	0.68 (0.01, 94.98)	0.73 (0.01, 106.83)	1.03 (0.01, 154.87)	Nilotinib 400mg BID	1.63 (0.02, 141.02)	1.20 (0.01, 199.42)	1.34 (0.01, 221.67)	0.05 (0.00, 10.19)
0.43 (0.04, 5.84)	0.46 (0.00, 129.13)	0.53 (0.02, 14.15)	0.43 (0.01, 29.32)	0.47 (0.01, 29.05)	0.64 (0.01, 49.30)	0.61 (0.01, 54.53)	Dasatinib 100mg QD	0.76 (0.01, 55.63)	0.85 (0.01, 63.54)	0.03 (0.00, 2.88)
0.60 (0.02, 18.74)	0.64 (0.00, 258.37)	0.73 (0.01, 38.32)	0.59 (0.00, 68.01)	0.63 (0.00, 72.94)	0.85 (0.01, 109.96)	0.84 (0.01, 120.89)	1.31 (0.02, 102.37)	Bosutinib 400mg QD	1.16 (0.01, 145.60)	0.04 (0.00, 7.31)
0.52 (0.02, 17.95)	0.54 (0.00, 257.95)	0.61 (0.01, 34.53)	0.52 (0.00, 59.85)	0.53 (0.00, 66.24)	0.74 (0.01, 108.62)	0.75 (0.00, 112.66)	1.18 (0.02, 87.45)	0.86 (0.01, 133.36)	Bosutinib 500mg QD	0.04 (0.00, 6.10)
13.73 (0.34, 932.45)	14.87 (0.03, 9762.19)	17.01 (0.39, 1280.81)	13.44 (0.24, 1418.34)	14.68 (0.25, 1338.55)	20.66 (0.12, 4662.81)	20.10 (0.10, 4702.10)	31.57 (0.35, 4469.55)	24.79 (0.14, 6757.74)	27.25 (0.16, 6507.19)	Imatinib 400mg QD after IFN-α

(e) Overall discontinuation

Imatinib 400mg QD	0.88 (0.19, 3.93)	1.51 (0.45, 5.00)	3.27 (0.36, 27.26)	7.40 (1.67, 31.59)	2.48 (0.61, 9.48)	1.00 (0.24, 4.27)	1.11 (0.29, 4.04)	0.81 (0.29, 2.47)	0.62 (0.07, 5.71)	1.80 (0.20, 15.76)	0.78 (0.10, 6.02)	1.64 (0.20, 13.52)	2.43 (0.28, 21.28)
1.14 (0.25, 5.36)	Imatinib 600mg QD	1.73 (0.27, 11.55)	3.64 (0.26, 52.14)	8.53 (1.40, 53.60)	2.80 (0.49, 16.27)	1.15 (0.16, 8.75)	1.27 (0.25, 6.28)	0.92 (0.15, 6.11)	0.72 (0.05, 10.65)	2.07 (0.15, 30.98)	0.87 (0.06, 11.75)	1.86 (0.14, 26.05)	2.80 (0.18, 41.42)
0.66 (0.20, 2.20)	0.58 (0.09, 3.71)	Imatinib 800mg QD	2.12 (0.16, 25.24)	4.89 (0.75, 31.65)	1.61 (0.31, 7.76)	0.66 (0.10, 4.22)	0.72 (0.13, 4.12)	0.53 (0.11, 2.71)	0.41 (0.03, 5.30)	1.18 (0.09, 13.84)	0.50 (0.05, 5.67)	1.05 (0.09, 13.13)	1.60 (0.13, 19.68)
0.31 (0.04, 2.79)	0.28 (0.02, 3.83)	0.47 (0.04, 6.07)	IFN + Ara-C	2.34 (0.16, 32.78)	0.77 (0.06, 9.44)	0.31 (0.02, 4.31)	0.34 (0.03, 4.66)	0.25 (0.02, 3.14)	0.20 (0.01, 4.20)	0.55 (0.03, 12.03)	0.23 (0.01, 4.88)	0.50 (0.02, 10.86)	0.76 (0.04, 16.99)
0.14 (0.03, 0.60)	0.12 (0.02, 0.72)	0.20 (0.03, 1.33)	0.43 (0.03, 6.10)	Imatinib 400mg QD + Ara-C	0.33 (0.06, 1.92)	0.13 (0.02, 1.08)	0.15 (0.02, 0.97)	0.11 (0.02, 0.70)	0.08 (0.01, 1.25)	0.24 (0.02, 3.58)	0.10 (0.01, 1.34)	0.22 (0.02, 2.95)	0.33 (0.02, 4.75)
0.40 (0.11, 1.63)	0.36 (0.06, 2.04)	0.62 (0.13, 3.19)	1.30 (0.11, 16.70)	2.99 (0.52, 17.95)	Imatinib 400mg QD + IFN	0.41 (0.06, 2.99)	0.44 (0.07, 2.70)	0.33 (0.06, 1.93)	0.26 (0.02, 3.58)	0.73 (0.06, 9.70)	0.31 (0.03, 3.90)	0.65 (0.05, 8.55)	0.98 (0.08, 13.83)
1.00 (0.23, 4.20)	0.87 (0.11, 6.39)	1.53 (0.24, 10.09)	3.25 (0.23, 43.22)	7.41 (0.92, 55.09)	2.47 (0.33, 16.18)	Nilotinib 300mg BID	1.10 (0.21, 5.80)	0.81 (0.13, 5.12)	0.62 (0.04, 8.92)	1.80 (0.13, 23.63)	0.77 (0.06, 8.75)	1.62 (0.12, 20.69)	2.43 (0.19, 32.41)
0.90 (0.25, 3.42)	0.79 (0.16, 3.93)	1.38 (0.24, 7.85)	2.91 (0.21, 37.00)	6.78 (1.03, 44.84)	2.25 (0.37, 13.74)	0.91 (0.17, 4.81)	Nilotinib 400mg BID	0.74 (0.14, 4.36)	0.56 (0.04, 7.61)	1.64 (0.12, 19.86)	0.69 (0.06, 8.19)	1.49 (0.12, 17.66)	2.18 (0.18, 28.61)
1.24 (0.40, 3.46)	1.08 (0.16, 6.65)	1.87 (0.37, 8.80)	3.93 (0.32, 43.34)	9.13 (1.44, 55.16)	3.01 (0.52, 16.56)	1.23 (0.20, 7.52)	1.35 (0.23, 7.28)	Dasatinib 100mg QD	0.78 (0.07, 9.14)	2.20 (0.19, 24.92)	0.94 (0.09, 9.74)	1.99 (0.19, 21.76)	3.00 (0.26, 33.84)
1.61 (0.18, 14.74)	1.38 (0.09, 20.35)	2.42 (0.19, 31.38)	5.10 (0.24, 113.58)	11.96 (0.80, 169.67)	3.90 (0.28, 54.59)	1.61 (0.11, 23.09)	1.78 (0.13, 23.73)	1.29 (0.11, 15.13)	Radotinib 300mg BID	2.80 (0.32, 26.49)	1.21 (0.06, 24.09)	2.61 (0.12, 56.10)	3.84 (0.18, 87.89)
0.55 (0.06, 4.94)	0.48 (0.03, 6.69)	0.85 (0.07, 10.74)	1.82 (0.08, 36.39)	4.17 (0.28, 56.93)	1.38 (0.10, 17.29)	0.56 (0.04, 7.92)	0.61 (0.05, 8.16)	0.45 (0.04, 5.31)	0.36 (0.04, 3.13)	Radotinib 400mg BID	0.42 (0.02, 8.42)	0.91 (0.04, 18.97)	1.38 (0.06, 26.60)
1.29 (0.17, 10.48)	1.15 (0.09, 15.41)	2.00 (0.18, 21.72)	4.26 (0.20, 90.70)	9.57 (0.75, 119.92)	3.20 (0.26, 38.52)	1.30 (0.11, 16.76)	1.44 (0.12, 17.92)	1.06 (0.10, 11.30)	0.82 (0.04, 17.80)	2.36 (0.12, 49.39)	Bosutinib 400mg QD	2.14 (0.11, 43.17)	3.23 (0.15, 64.24)
0.61 (0.07, 5.00)	0.54 (0.04, 7.19)	0.95 (0.08, 11.01)	1.98 (0.09, 42.11)	4.51 (0.34, 58.19)	1.53 (0.12, 18.28)	0.62 (0.05, 8.28)	0.67 (0.06, 8.17)	0.50 (0.05, 5.23)	0.38 (0.02, 8.02)	1.10 (0.05, 22.70)	0.47 (0.02, 9.25)	Bosutinib 500mg QD	1.49 (0.07, 33.32)
0.41 (0.05, 3.58)	0.36 (0.02, 5.42)	0.62 (0.05, 7.53)	1.31 (0.06, 26.84)	3.04 (0.21, 41.09)	1.02 (0.07, 13.04)	0.41 (0.03, 5.40)	0.46 (0.03, 5.69)	0.33 (0.03, 3.82)	0.26 (0.01, 5.64)	0.73 (0.04, 16.26)	0.31 (0.02, 6.62)	0.67 (0.03, 14.24)	Imatinib 400mg QD after IFN-α

(f) Discontinuation for drug-related AEs

Imatinib 400mg QD	1.04 (0.37, 3.25)	2.32 (1.00, 5.25)	3.25 (0.85, 12.64)	4.94 (1.68, 12.42)	14.05 (4.15, 45.81)	1.28 (0.49, 3.88)	2.01 (0.94, 5.50)	1.42 (0.71, 2.81)	1.54 (0.30, 9.48)	4.14 (0.92, 21.57)	1.53 (0.43, 5.60)	4.18 (1.23, 16.04)	8.86 (1.48, 87.98)
0.97 (0.31, 2.73)	Imatinib 600mg QD	2.23 (0.57, 8.59)	3.13 (0.56, 16.64)	4.82 (1.27, 13.22)	13.59 (3.64, 45.20)	1.23 (0.31, 5.34)	1.94 (0.65, 6.77)	1.36 (0.35, 4.64)	1.49 (0.20, 11.59)	3.98 (0.59, 28.95)	1.47 (0.28, 7.50)	4.10 (0.71, 21.04)	8.46 (0.98, 104.39)
0.43 (0.19, 1.00)	0.45 (0.12, 1.76)	Imatinib 800mg QD	1.41 (0.29, 7.02)	2.14 (0.55, 7.18)	6.09 (1.37, 25.05)	0.55 (0.16, 2.27)	0.86 (0.29, 3.17)	0.61 (0.21, 1.77)	0.66 (0.11, 4.68)	1.79 (0.32, 11.04)	0.66 (0.15, 2.97)	1.82 (0.39, 9.21)	3.82 (0.54, 45.38)
0.31 (0.08, 1.17)	0.32 (0.06, 1.77)	0.71 (0.14, 3.43)	IFN + Ara-C	1.53 (0.26, 7.09)	4.34 (0.70, 25.58)	0.39 (0.08, 2.36)	0.62 (0.14, 3.32)	0.43 (0.09, 1.95)	0.47 (0.06, 4.88)	1.27 (0.16, 10.94)	0.47 (0.07, 3.10)	1.32 (0.20, 8.18)	2.77 (0.28, 38.49)
0.20 (0.08, 0.60)	0.21 (0.08, 0.78)	0.47 (0.14, 1.81)	0.66 (0.14, 3.89)	Imatinib 400mg QD + Ara-C	2.80 (0.96, 10.34)	0.26 (0.07, 1.27)	0.40 (0.14, 1.75)	0.29 (0.09, 1.07)	0.31 (0.05, 2.61)	0.85 (0.15, 6.28)	0.31 (0.07, 1.71)	0.85 (0.18, 4.79)	1.81 (0.24, 23.29)
0.07 (0.02, 0.24)	0.07 (0.02, 0.27)	0.16 (0.04, 0.73)	0.23 (0.04, 1.44)	0.36 (0.10, 1.04)	Imatinib 400mg QD + IFN	0.09 (0.02, 0.50)	0.14 (0.04, 0.66)	0.10 (0.02, 0.41)	0.11 (0.01, 0.97)	0.30 (0.04, 2.28)	0.11 (0.02, 0.65)	0.30 (0.05, 1.75)	0.63 (0.07, 8.06)
0.78 (0.26, 2.03)	0.81 (0.19, 3.24)	1.81 (0.44, 6.40)	2.55 (0.42, 13.30)	3.90 (0.79, 13.75)	11.02 (2.01, 47.73)	Nilotinib 300mg BID	1.56 (0.54, 4.89)	1.11 (0.29, 3.55)	1.21 (0.16, 9.21)	3.24 (0.49, 21.49)	1.19 (0.22, 5.52)	3.34 (0.60, 15.91)	6.80 (0.82, 87.32)
0.50 (0.18, 1.07)	0.52 (0.15, 1.54)	1.16 (0.32, 3.42)	1.62 (0.30, 7.23)	2.48 (0.57, 7.38)	7.07 (1.51, 26.09)	0.64 (0.20, 1.87)	Nilotinib 400mg BID	0.71 (0.20, 1.90)	0.78 (0.11, 5.33)	2.07 (0.33, 12.57)	0.77 (0.14, 3.23)	2.12 (0.41, 8.98)	4.32 (0.54, 48.99)
0.70 (0.36, 1.41)	0.73 (0.22, 2.84)	1.64 (0.57, 4.83)	2.31 (0.51, 11.21)	3.50 (0.94, 10.59)	9.89 (2.42, 40.98)	0.90 (0.28, 3.41)	1.41 (0.53, 5.01)	Dasatinib 100mg QD	1.09 (0.19, 7.68)	2.97 (0.56, 17.20)	1.08 (0.25, 4.60)	2.97 (0.71, 13.61)	6.29 (0.93, 73.09)
0.65 (0.11, 3.32)	0.67 (0.09, 4.88)	1.51 (0.21, 9.42)	2.15 (0.21, 17.07)	3.26 (0.38, 19.24)	9.24 (1.03, 70.63)	0.83 (0.11, 6.09)	1.29 (0.19, 9.15)	0.92 (0.13, 5.39)	Radotinib 300mg BID	2.65 (0.61, 12.05)	0.99 (0.12, 8.04)	2.70 (0.33, 21.78)	5.75 (0.47, 95.73)
0.24 (0.05, 1.09)	0.25 (0.03, 1.69)	0.56 (0.09, 3.14)	0.79 (0.09, 6.12)	1.18 (0.16, 6.83)	3.34 (0.44, 23.27)	0.31 (0.05, 2.06)	0.48 (0.08, 3.00)	0.34 (0.06, 1.80)	0.38 (0.08, 1.63)	Radotinib 400mg BID	0.37 (0.05, 2.62)	1.01 (0.14, 7.37)	2.14 (0.19, 32.08)
0.65 (0.18, 2.35)	0.68 (0.13, 3.56)	1.52 (0.34, 6.78)	2.11 (0.32, 13.81)	3.22 (0.58, 14.37)	9.04 (1.54, 51.21)	0.84 (0.18, 4.59)	1.31 (0.31, 6.98)	0.93 (0.22, 3.93)	1.01 (0.12, 8.68)	2.73 (0.38, 20.51)	Bosutinib 400mg QD	2.76 (0.45, 17.06)	5.64 (0.64, 78.19)
0.24 (0.06, 0.81)	0.24 (0.05, 1.40)	0.55 (0.11, 2.54)	0.76 (0.12, 4.94)	1.18 (0.21, 5.44)	3.28 (0.57, 18.97)	0.30 (0.06, 1.67)	0.47 (0.11, 2.43)	0.34 (0.07, 1.40)	0.37 (0.05, 3.05)	0.99 (0.14, 7.35)	0.36 (0.06, 2.23)	Bosutinib 500mg QD	2.05 (0.24, 30.14)
0.11 (0.01, 0.67)	0.12 (0.01, 1.02)	0.26 (0.02, 1.86)	0.36 (0.03, 3.52)	0.55 (0.04, 4.20)	1.59 (0.12, 13.90)	0.15 (0.01, 1.22)	0.23 (0.02, 1.87)	0.16 (0.01, 1.07)	0.17 (0.01, 2.15)	0.47 (0.03, 5.25)	0.18 (0.01, 1.57)	0.49 (0.03, 4.19)	Ponatinib 45mg QD

Appendix 1 WinBUGS codes

"A" to "O" refer to each treatment.

A: Imatinib 400mg QD; B: Imatinib 600mg QD; C: Imatinib 800mg QD; D: IFN-α + Ara-C; E: Imatinib 400mg QD + Ara-C; F: Imatinib 400mg QD + IFN-α; G: Nilotinib 300mg BID; H: Nilotinib 400mg BID; I: Dasatinib 100mg QD; J: Radotinib 300mg BID; K: Radotinib 400mg BID; L: Bosutinib 400mg QD; M: Bosutinib 500mg QD; N: Ponatinib 45mg QD; O: Imatinib 400mg QD after IFN-α.

For example: Consistency model for "MMR within 12 months"

```
model {
	for (i in 1:ns) {
		# Likelihood for each arm
		for (k in 1:na[i]) {
			r[i, k] ~ dbin(p[i, k], n[i, k])
			logit(p[i, k]) <- mu[i] + delta[i, k]
		}

		# Study-level relative effects
		# The arms are given in the order (arm_1, arm_2, ..., arm_{n_a-1}, arm_{n_a}).
		# The relative effects are parameterized as d[arm_1, arm_k].
		w[i, 1] <- 0
		delta[i, 1] <- 0
		for (k in 2:na[i]) { # parameterize multi-arm trials using a trick to avoid dmnorm
			delta[i, k] ~ dnorm(md[i, k], taud[i, k])
			md[i, k] <- d[t[i, 1], t[i, k]] + sw[i, k]
			taud[i, k] <- tau.d * 2 * (k - 1) / k
			w[i, k] <- delta[i, k] - d[t[i, 1], t[i, k]]
			sw[i, k] <- sum(w[i, 1:k-1]) / (k - 1)
		}
	}

	# Relative effect matrix
	d[1,1] <- 0
	d[1,2] <- d.A.B
	d[1,3] <- d.A.C
	d[1,4] <- d.A.D
	d[1,5] <- d.A.E
	d[1,6] <- d.A.F
	d[1,7] <- d.A.G
	d[1,8] <- d.A.H
	d[1,9] <- d.A.I
	d[1,10] <- d.A.J
	d[1,11] <- d.A.K
```

```
d[1,12] <- d.A.L
d[1,13] <- d.A.M
d[1,14] <- d.A.N
d[1,15] <- d.A.O
d[2,1] <- -d.A.B
d[2,2] <- 0
d[2,3] <- -d.A.B + d.A.C
d[2,4] <- -d.A.B + d.A.D
d[2,5] <- -d.A.B + d.A.E
d[2,6] <- -d.A.B + d.A.F
d[2,7] <- -d.A.B + d.A.G
d[2,8] <- -d.A.B + d.A.H
d[2,9] <- -d.A.B + d.A.I
d[2,10] <- -d.A.B + d.A.J
d[2,11] <- -d.A.B + d.A.K
d[2,12] <- -d.A.B + d.A.L
d[2,13] <- -d.A.B + d.A.M
d[2,14] <- -d.A.B + d.A.N
d[2,15] <- -d.A.B + d.A.O
d[3,1] <- -d.A.C
d[3,2] <- d.A.B + -d.A.C
d[3,3] <- 0
d[3,4] <- -d.A.C + d.A.D
d[3,5] <- -d.A.C + d.A.E
d[3,6] <- -d.A.C + d.A.F
d[3,7] <- -d.A.C + d.A.G
d[3,8] <- -d.A.C + d.A.H
d[3,9] <- -d.A.C + d.A.I
d[3,10] <- -d.A.C + d.A.J
d[3,11] <- -d.A.C + d.A.K
d[3,12] <- -d.A.C + d.A.L
d[3,13] <- -d.A.C + d.A.M
d[3,14] <- -d.A.C + d.A.N
d[3,15] <- -d.A.C + d.A.O
d[4,1] <- -d.A.D
d[4,2] <- d.A.B + -d.A.D
d[4,3] <- d.A.C + -d.A.D
d[4,4] <- 0
d[4,5] <- -d.A.D + d.A.E
d[4,6] <- -d.A.D + d.A.F
d[4,7] <- -d.A.D + d.A.G
d[4,8] <- -d.A.D + d.A.H
d[4,9] <- -d.A.D + d.A.I
d[4,10] <- -d.A.D + d.A.J
```

```
d[4,11] <- -d.A.D + d.A.K
d[4,12] <- -d.A.D + d.A.L
d[4,13] <- -d.A.D + d.A.M
d[4,14] <- -d.A.D + d.A.N
d[4,15] <- -d.A.D + d.A.O
d[5,1] <- -d.A.E
d[5,2] <- d.A.B + -d.A.E
d[5,3] <- d.A.C + -d.A.E
d[5,4] <- d.A.D + -d.A.E
d[5,5] <- 0
d[5,6] <- -d.A.E + d.A.F
d[5,7] <- -d.A.E + d.A.G
d[5,8] <- -d.A.E + d.A.H
d[5,9] <- -d.A.E + d.A.I
d[5,10] <- -d.A.E + d.A.J
d[5,11] <- -d.A.E + d.A.K
d[5,12] <- -d.A.E + d.A.L
d[5,13] <- -d.A.E + d.A.M
d[5,14] <- -d.A.E + d.A.N
d[5,15] <- -d.A.E + d.A.O
d[6,1] <- -d.A.F
d[6,2] <- d.A.B + -d.A.F
d[6,3] <- d.A.C + -d.A.F
d[6,4] <- d.A.D + -d.A.F
d[6,5] <- d.A.E + -d.A.F
d[6,6] <- 0
d[6,7] <- -d.A.F + d.A.G
d[6,8] <- -d.A.F + d.A.H
d[6,9] <- -d.A.F + d.A.I
d[6,10] <- -d.A.F + d.A.J
d[6,11] <- -d.A.F + d.A.K
d[6,12] <- -d.A.F + d.A.L
d[6,13] <- -d.A.F + d.A.M
d[6,14] <- -d.A.F + d.A.N
d[6,15] <- -d.A.F + d.A.O
d[7,1] <- -d.A.G
d[7,2] <- d.A.B + -d.A.G
d[7,3] <- d.A.C + -d.A.G
d[7,4] <- d.A.D + -d.A.G
d[7,5] <- d.A.E + -d.A.G
d[7,6] <- d.A.F + -d.A.G
d[7,7] <- 0
d[7,8] <- -d.A.G + d.A.H
d[7,9] <- -d.A.G + d.A.I
```

```
d[7,10] <- -d.A.G + d.A.J
d[7,11] <- -d.A.G + d.A.K
d[7,12] <- -d.A.G + d.A.L
d[7,13] <- -d.A.G + d.A.M
d[7,14] <- -d.A.G + d.A.N
d[7,15] <- -d.A.G + d.A.O
d[8,1] <- -d.A.H
d[8,2] <- d.A.B + -d.A.H
d[8,3] <- d.A.C + -d.A.H
d[8,4] <- d.A.D + -d.A.H
d[8,5] <- d.A.E + -d.A.H
d[8,6] <- d.A.F + -d.A.H
d[8,7] <- d.A.G + -d.A.H
d[8,8] <- 0
d[8,9] <- -d.A.H + d.A.I
d[8,10] <- -d.A.H + d.A.J
d[8,11] <- -d.A.H + d.A.K
d[8,12] <- -d.A.H + d.A.L
d[8,13] <- -d.A.H + d.A.M
d[8,14] <- -d.A.H + d.A.N
d[8,15] <- -d.A.H + d.A.O
d[9,1] <- -d.A.I
d[9,2] <- d.A.B + -d.A.I
d[9,3] <- d.A.C + -d.A.I
d[9,4] <- d.A.D + -d.A.I
d[9,5] <- d.A.E + -d.A.I
d[9,6] <- d.A.F + -d.A.I
d[9,7] <- d.A.G + -d.A.I
d[9,8] <- d.A.H + -d.A.I
d[9,9] <- 0
d[9,10] <- -d.A.I + d.A.J
d[9,11] <- -d.A.I + d.A.K
d[9,12] <- -d.A.I + d.A.L
d[9,13] <- -d.A.I + d.A.M
d[9,14] <- -d.A.I + d.A.N
d[9,15] <- -d.A.I + d.A.O
d[10,1] <- -d.A.J
d[10,2] <- d.A.B + -d.A.J
d[10,3] <- d.A.C + -d.A.J
d[10,4] <- d.A.D + -d.A.J
d[10,5] <- d.A.E + -d.A.J
d[10,6] <- d.A.F + -d.A.J
d[10,7] <- d.A.G + -d.A.J
d[10,8] <- d.A.H + -d.A.J
```

```
d[10,9] <- d.A.I + -d.A.J
d[10,10] <- 0
d[10,11] <- -d.A.J + d.A.K
d[10,12] <- -d.A.J + d.A.L
d[10,13] <- -d.A.J + d.A.M
d[10,14] <- -d.A.J + d.A.N
d[10,15] <- -d.A.J + d.A.O
d[11,1] <- -d.A.K
d[11,2] <- d.A.B + -d.A.K
d[11,3] <- d.A.C + -d.A.K
d[11,4] <- d.A.D + -d.A.K
d[11,5] <- d.A.E + -d.A.K
d[11,6] <- d.A.F + -d.A.K
d[11,7] <- d.A.G + -d.A.K
d[11,8] <- d.A.H + -d.A.K
d[11,9] <- d.A.I + -d.A.K
d[11,10] <- d.A.J + -d.A.K
d[11,11] <- 0
d[11,12] <- -d.A.K + d.A.L
d[11,13] <- -d.A.K + d.A.M
d[11,14] <- -d.A.K + d.A.N
d[11,15] <- -d.A.K + d.A.O
d[12,1] <- -d.A.L
d[12,2] <- d.A.B + -d.A.L
d[12,3] <- d.A.C + -d.A.L
d[12,4] <- d.A.D + -d.A.L
d[12,5] <- d.A.E + -d.A.L
d[12,6] <- d.A.F + -d.A.L
d[12,7] <- d.A.G + -d.A.L
d[12,8] <- d.A.H + -d.A.L
d[12,9] <- d.A.I + -d.A.L
d[12,10] <- d.A.J + -d.A.L
d[12,11] <- d.A.K + -d.A.L
d[12,12] <- 0
d[12,13] <- -d.A.L + d.A.M
d[12,14] <- -d.A.L + d.A.N
d[12,15] <- -d.A.L + d.A.O
d[13,1] <- -d.A.M
d[13,2] <- d.A.B + -d.A.M
d[13,3] <- d.A.C + -d.A.M
d[13,4] <- d.A.D + -d.A.M
d[13,5] <- d.A.E + -d.A.M
d[13,6] <- d.A.F + -d.A.M
d[13,7] <- d.A.G + -d.A.M
```

```
d[13,8] <- d.A.H + -d.A.M
d[13,9] <- d.A.I + -d.A.M
d[13,10] <- d.A.J + -d.A.M
d[13,11] <- d.A.K + -d.A.M
d[13,12] <- d.A.L + -d.A.M
d[13,13] <- 0
d[13,14] <- -d.A.M + d.A.N
d[13,15] <- -d.A.M + d.A.O
d[14,1] <- -d.A.N
d[14,2] <- d.A.B + -d.A.N
d[14,3] <- d.A.C + -d.A.N
d[14,4] <- d.A.D + -d.A.N
d[14,5] <- d.A.E + -d.A.N
d[14,6] <- d.A.F + -d.A.N
d[14,7] <- d.A.G + -d.A.N
d[14,8] <- d.A.H + -d.A.N
d[14,9] <- d.A.I + -d.A.N
d[14,10] <- d.A.J + -d.A.N
d[14,11] <- d.A.K + -d.A.N
d[14,12] <- d.A.L + -d.A.N
d[14,13] <- d.A.M + -d.A.N
d[14,14] <- 0
d[14,15] <- -d.A.N + d.A.O
d[15,1] <- -d.A.O
d[15,2] <- d.A.B + -d.A.O
d[15,3] <- d.A.C + -d.A.O
d[15,4] <- d.A.D + -d.A.O
d[15,5] <- d.A.E + -d.A.O
d[15,6] <- d.A.F + -d.A.O
d[15,7] <- d.A.G + -d.A.O
d[15,8] <- d.A.H + -d.A.O
d[15,9] <- d.A.I + -d.A.O
d[15,10] <- d.A.J + -d.A.O
d[15,11] <- d.A.K + -d.A.O
d[15,12] <- d.A.L + -d.A.O
d[15,13] <- d.A.M + -d.A.O
d[15,14] <- d.A.N + -d.A.O
d[15,15] <- 0

# Study baseline priors
for (i in 1:ns) {
	mu[i] ~ dnorm(0, 1.769E-4)
}
```

```
# Variance prior
sd.d ~ dunif(0, 5.012E0)
tau.d <- pow(sd.d, -2)

# Effect parameter priors
d.A.B ~ dnorm(0, 1.769E-4)
d.A.C ~ dnorm(0, 1.769E-4)
d.A.D ~ dnorm(0, 1.769E-4)
d.A.E ~ dnorm(0, 1.769E-4)
d.A.F ~ dnorm(0, 1.769E-4)
d.A.G ~ dnorm(0, 1.769E-4)
d.A.H ~ dnorm(0, 1.769E-4)
d.A.I ~ dnorm(0, 1.769E-4)
d.A.J ~ dnorm(0, 1.769E-4)
d.A.K ~ dnorm(0, 1.769E-4)
d.A.L ~ dnorm(0, 1.769E-4)
d.A.M ~ dnorm(0, 1.769E-4)
d.A.N ~ dnorm(0, 1.769E-4)
```
