# Supplementary material for: Comparative efficacy and tolerability of front-line treatments for newly diagnosed chronic-phase chronic myeloid leukemia: an update network meta-analysis
Source: BMC Cancer. 2019 Aug 28;19:849. doi: 10.1186/s12885-019-6039-9 (PMC6714291; doi:10.1186/s12885-019-6039-9)

Additional file 2:

Figure S1 Risk of bias assessment (a) Risk of bias graph; (b) Risk of bias summary.

Figure S2 Rankograms for six outcomes (a) MMR within 12 months; (b) CCyR within 12 months; (c) Progression to AP-CML; (d) Serious AEs; (e) Overall discontinuation; (f) Discontinuation for drug-related AEs.

Figure S3 Funnel plots for six outcomes (a) MMR within 12 months; (b) CCyR within 12 months; (c) Progression to AP-CML; (d) Serious AEs; (e) Overall discontinuation; (f) Discontinuation for drug-related AEs.

Figure S1 Risk of bias assessment

(a) Risk of bias graph

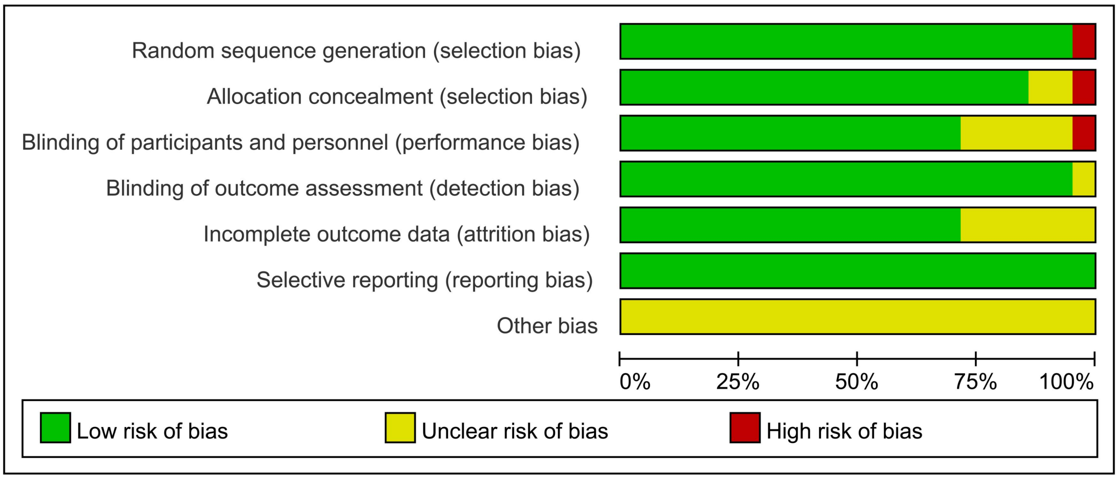

(b) Risk of bias summary

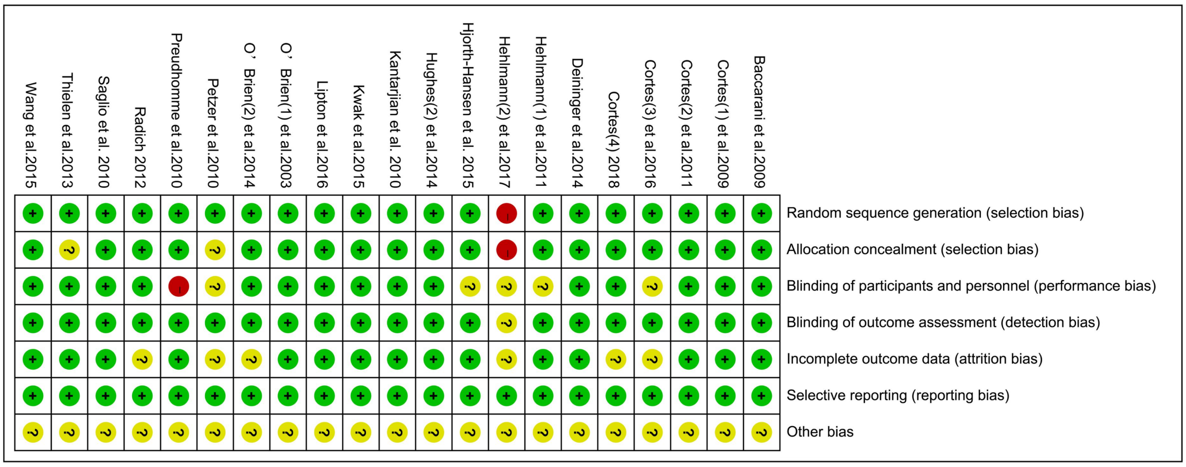

Figure S2 Rankograms for six outcomes

"A" to "O" refer to each treatment. A: Imatinib 400mg QD; B: Imatinib 600mg QD; C: Imatinib 800mg QD; D: IFN-α + Ara-C; E: Imatinib 400mg QD + Ara-C; F: Imatinib 400mg QD + IFN-α; G: Nilotinib 300mg BID; H: Nilotinib 400mg BID; I: Dasatinib 100mg QD; J: Radotinib 300mg BID; K: Radotinib 400mg BID; L: Bosutinib 400mg QD; M: Bosutinib 500mg QD; N: Ponatinib 45mg QD; O: Imatinib 400mg QD after IFN-α.

(a) MMR within 12 months

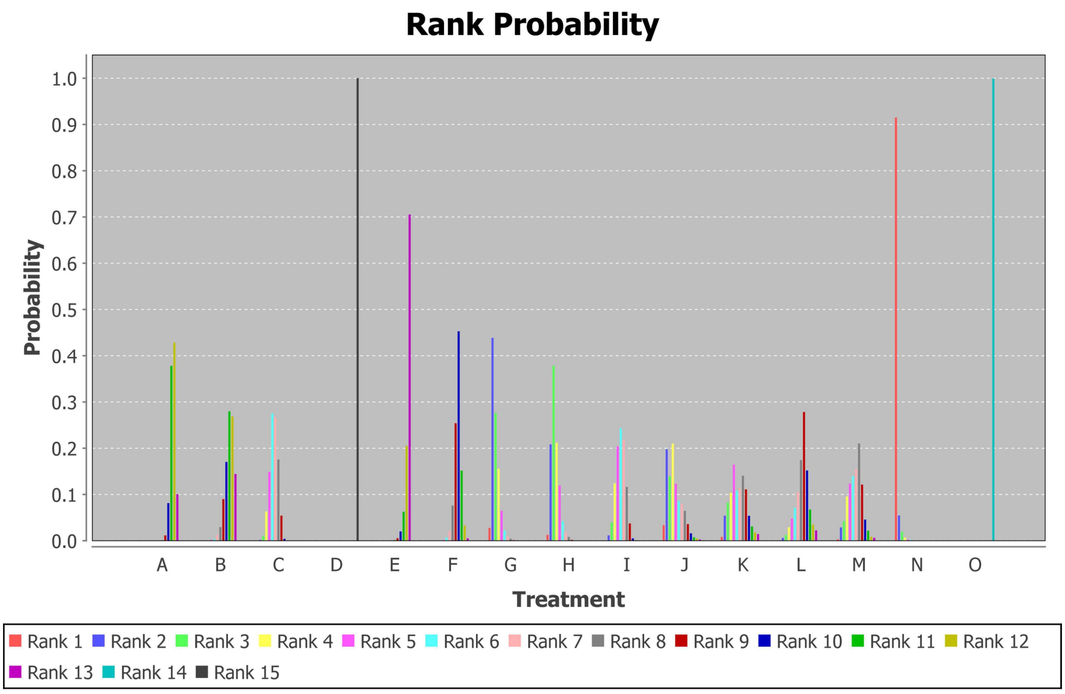

(b) CCyR within 12 months

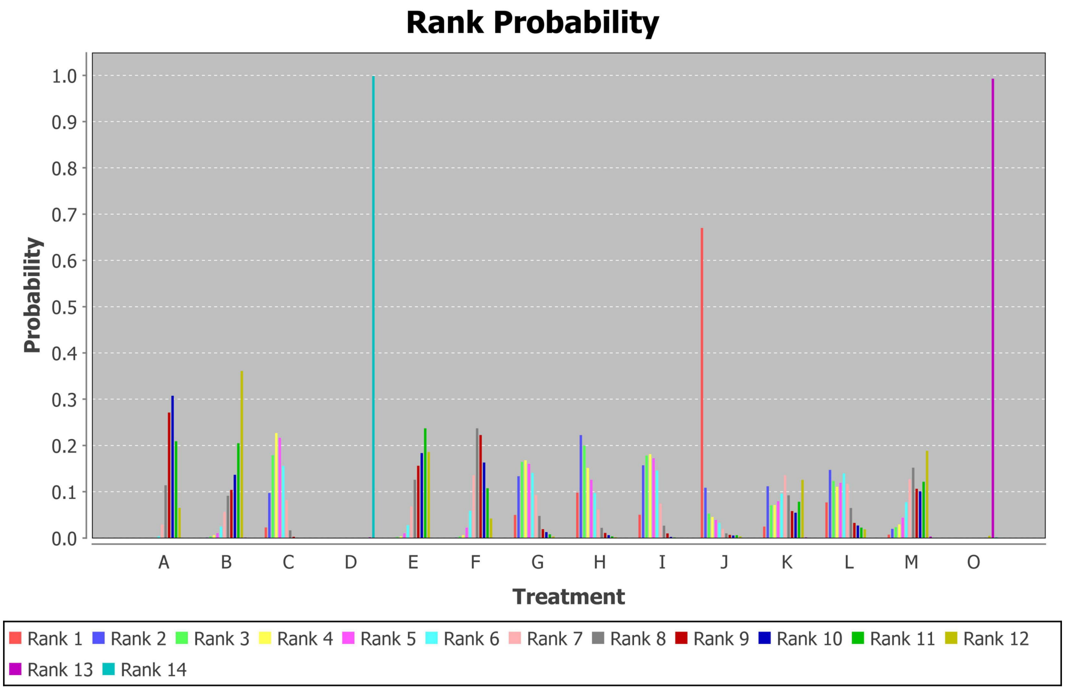

(c) Progression to AP-CML

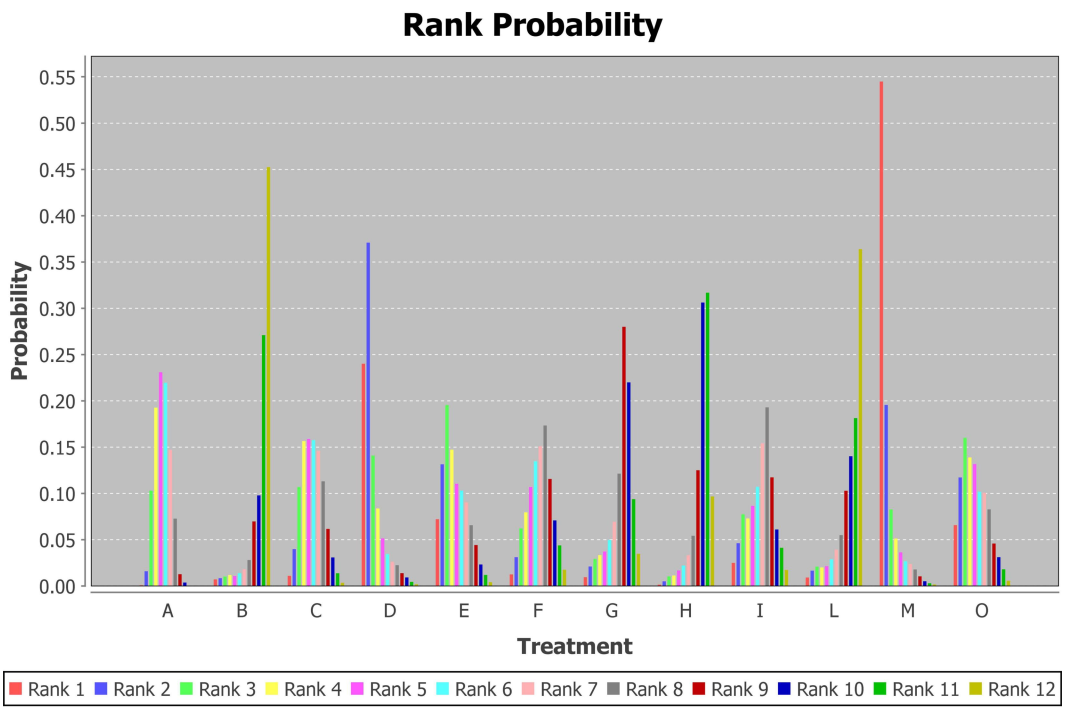

(d) Serious AEs

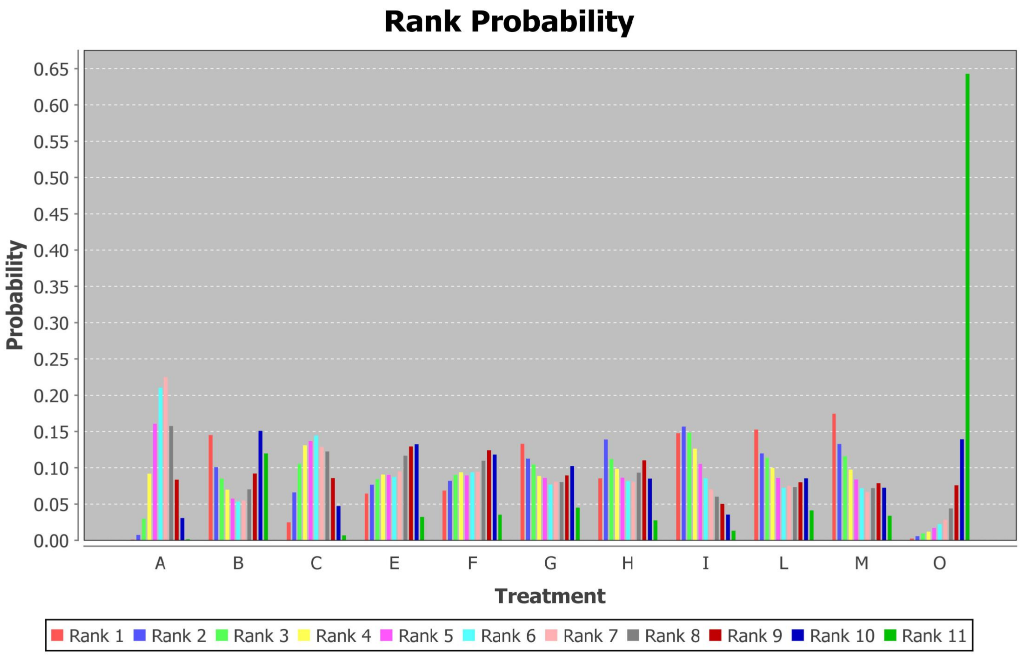

(e) Overall discontinuation

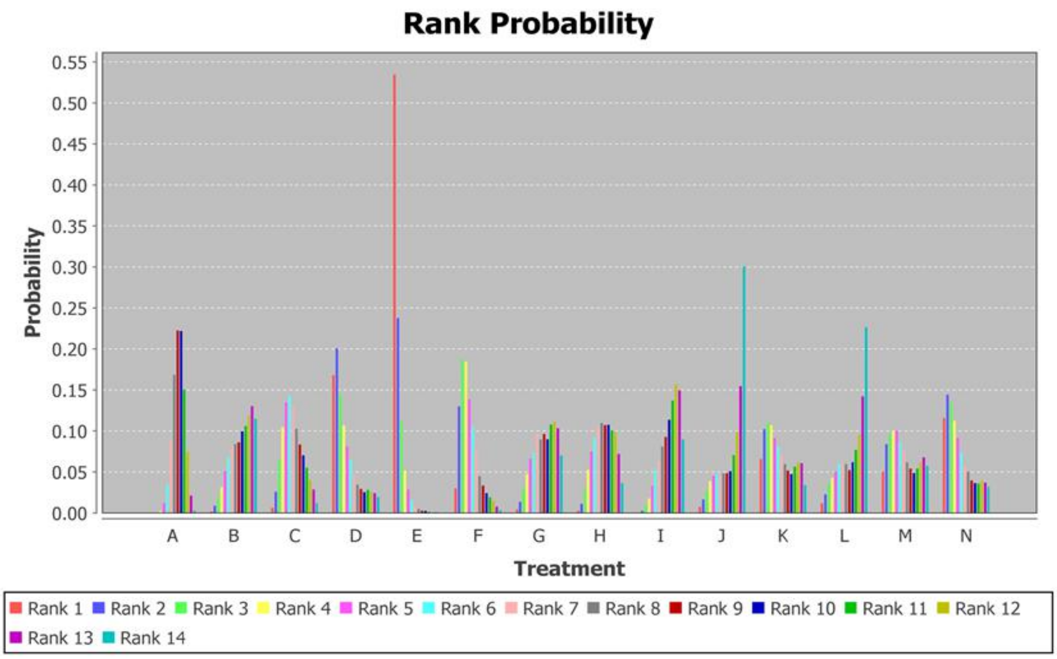

(f) Discontinuation for drug-related AEs

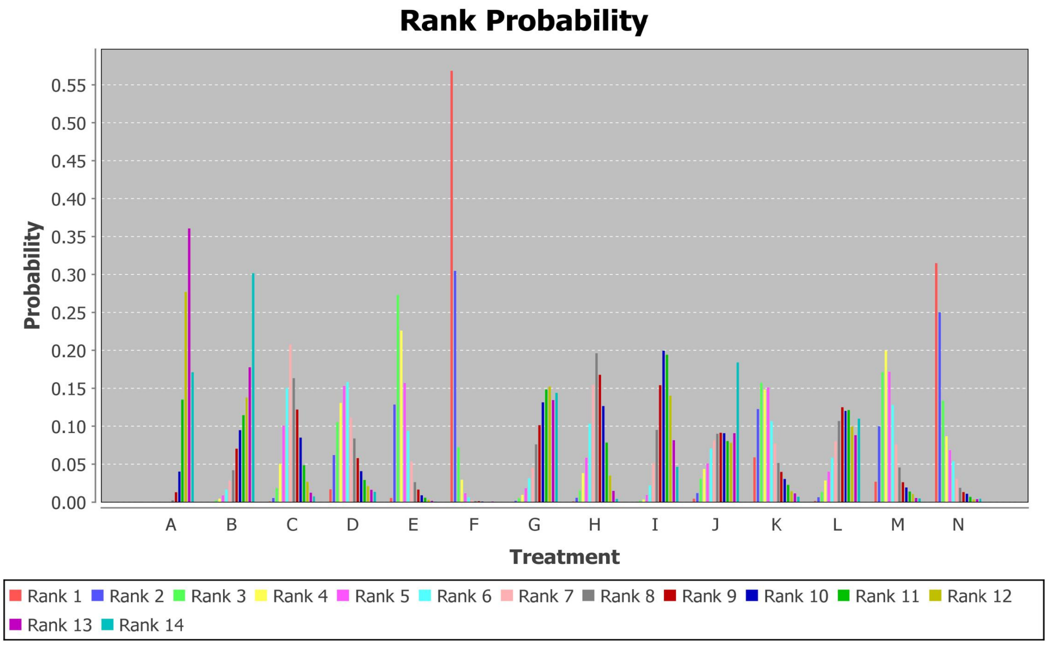

Figure S3 Funnel plots for six outcomes

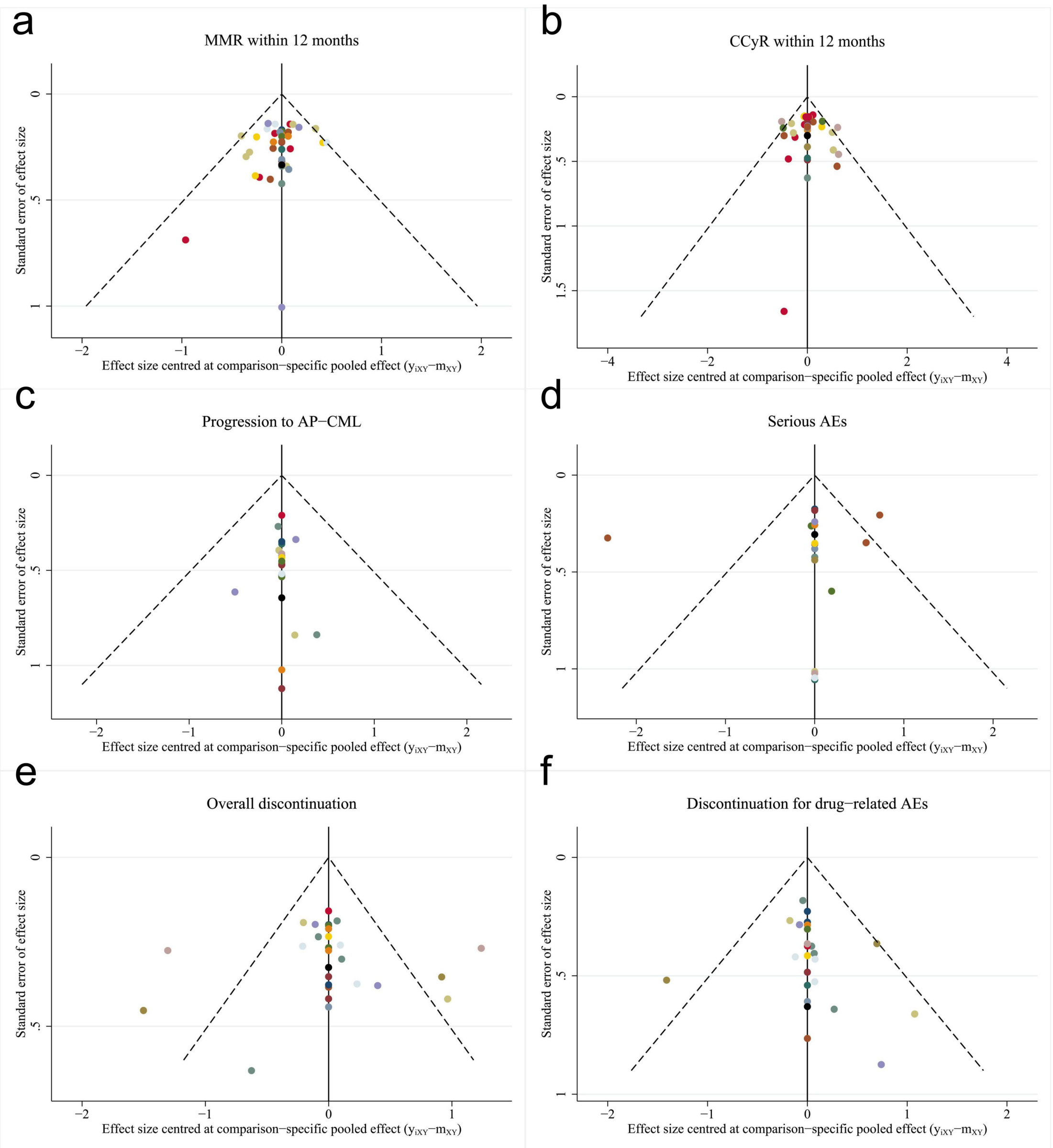

Supplement: Supplementary file 2 — Figure S1. Risk of bias assessment (a) Risk of bias graph; (b) Risk of bias summary. Figure S2. Rankograms for six outcomes (a) MMR within 12 months; (b) CCyR within 12 months; (c) Progression to AP-CML; (d) Serious AEs; (e) Overall discontinuation; (f) Discontinuation for drug-related AEs. Figure S3. Funnel plots for six outcomes (a) MMR within 12 months; (b) CCyR within 12 months; (c) Progression to AP-CML; (d) Serious AEs; (e) Overall discontinuation; (f) Discontinuation for drug-related AEs (PDF 2981 kb) [file 12885_2019_6039_MOESM2_ESM.pdf]
